# Genetic Architecture of Atherosclerosis in Mice: A Systems Genetics Analysis of Common Inbred Strains

**DOI:** 10.1371/journal.pgen.1005711

**Published:** 2015-12-22

**Authors:** Brian J. Bennett, Richard C. Davis, Mete Civelek, Luz Orozco, Judy Wu, Hannah Qi, Calvin Pan, René R. Sevag Packard, Eleazar Eskin, Mujing Yan, Todd Kirchgessner, Zeneng Wang, Xinmin Li, Jill C. Gregory, Stanley L. Hazen, Peter S. Gargalovic, Aldons J. Lusis

**Affiliations:** 1 Departments of Medicine, Human Genetics, and Microbiology, Immunology, and Molecular Genetics, University of California, Los Angeles, Los Angeles, California, United States of America; 2 Department of Computer Science, University of California, Los Angeles, Los Angeles, California, United States of America; 3 Department of Cardiovascular Drug Discovery, Bristol-Myers Squibb, Princeton, New Jersey, United States of America; 4 Department of Cellular and Molecular Medicine (NC10), Cleveland Clinic Lerner Research Institute, Cleveland, Ohio, United States of America; Stanford University School of Medicine, UNITED STATES

## Abstract

Common forms of atherosclerosis involve multiple genetic and environmental factors. While human genome-wide association studies have identified numerous loci contributing to coronary artery disease and its risk factors, these studies are unable to control environmental factors or examine detailed molecular traits in relevant tissues. We now report a study of natural variations contributing to atherosclerosis and related traits in over 100 inbred strains of mice from the Hybrid Mouse Diversity Panel (HMDP). The mice were made hyperlipidemic by transgenic expression of human apolipoprotein E-Leiden (APOE-Leiden) and human cholesteryl ester transfer protein (CETP). The mice were examined for lesion size and morphology as well as plasma lipid, insulin and glucose levels, and blood cell profiles. A subset of mice was studied for plasma levels of metabolites and cytokines. We also measured global transcript levels in aorta and liver. Finally, the uptake of acetylated LDL by macrophages from HMDP mice was quantitatively examined. Loci contributing to the traits were mapped using association analysis, and relationships among traits were examined using correlation and statistical modeling. A number of conclusions emerged. First, relationships among atherosclerosis and the risk factors in mice resemble those found in humans. Second, a number of trait-loci were identified, including some overlapping with previous human and mouse studies. Third, gene expression data enabled enrichment analysis of pathways contributing to atherosclerosis and prioritization of candidate genes at associated loci in both mice and humans. Fourth, the data provided a number of mechanistic inferences; for example, we detected no association between macrophage uptake of acetylated LDL and atherosclerosis. Fifth, broad sense heritability for atherosclerosis was much larger than narrow sense heritability, indicating an important role for gene-by-gene interactions. Sixth, stepwise linear regression showed that the combined variations in plasma metabolites, including LDL/VLDL-cholesterol, trimethylamine N-oxide (TMAO), arginine, glucose and insulin, account for approximately 30 to 40% of the variation in atherosclerotic lesion area. Overall, our data provide a rich resource for studies of complex interactions underlying atherosclerosis.

## Author Summary

While recent genetic association studies in human populations have succeeded in identifying genetic loci that contribute to coronary artery disease (CAD) and related phenotypes, these loci explain only a small fraction of the genetic variation in CAD and associated traits. Here, we present a complementary approach using association analysis of atherosclerotic traits among inbred strains of mice. A strength of this approach is that it enables in-depth phenotypic characterization including gene expression and metabolic profiling across a variety of tissues, and integration of these molecular phenotypes with coronary artery disease itself. A striking finding was the large fraction of atherosclerosis that was explained by genetic interactions. Association analysis allowed us to identify genetic loci for atherosclerotic lesion area as well as transcript, cytokine and metabolite levels, and relationships among the traits were examined by correlation and network modeling. The plasma metabolites associated with atherosclerosis in mice, namely, LDL/VLDL-cholesterol, TMAO, arginine, glucose and insulin, overlapped with those observed in humans and accounted for approximately 30 to 40% of the observed variation in atherosclerotic lesion area. In summary, our data provide a detailed overview of the genetic architecture of atherosclerosis in mice and a rich resource for studies of the complex genetic and metabolic interactions that underlie the disease.

## Introduction

Inheritance plays an important role in the pathogenesis of coronary artery disease (CAD), the leading cause of death in the developed world [1–4]. Recent genome-wide association studies (GWAS), involving hundreds of thousands of individuals have identified numerous loci contributing to CAD traits and to risk factors such as blood lipoprotein levels and blood pressure. A major challenge at present is to identify the causal genes at those loci and to understand the mechanisms by which they contribute to disease [5, 6]. Most of the loci identified do not contain known candidates; for example, data from nearly 200,000 people identified 46 genetic loci associated with CAD, but only 17 of these loci contain genes for known risk factors such as lipids and blood pressure [5]. In a few cases, novel loci contributing to CAD have been successfully dissected using a combination of human and experimental mouse studies [7–9]. Such GWAS and follow-up studies do, however, have some important limitations. In particular, they are poorly powered to examine gene-by-gene and environmental interactions or to identify rare variants. Consequently, for most traits that have been studied, even very large association studies explain a small fraction of the heritability of the traits [9, 10].

A complementary approach to studying common forms of CAD is to use naturally occurring variations in experimental organisms such as rats or mice. Important advantages include the ability to control the environment and to monitor both clinical and molecular phenotypes in detail. Over the past 20 years, quantitative trait locus (QTL) analysis has identified hundreds of loci for common disease traits. Unfortunately, this has led to the identification of relatively few genes and novel pathways, primarily because of the low resolution of linkage analysis [11]. However, with the recent sequencing of many mouse strains, it has become feasible to carry out high-resolution mapping in mice using association rather than linkage [12–15].

We now report association analysis of atherosclerosis and related traits in a population of over 100 common inbred strains, termed the Hybrid Mouse Diversity Panel (HMDP). The panel allows mapping of complex traits by association analysis, providing resolution at least an order of magnitude better than that achieved by traditional linkage analysis [12]. The approach has now been used to identify novel genes for a number of traits, including several examples in which the genes have been validated using transgenic models [13, 14, 16, 17]. Because the development of significant atherosclerotic lesions in mice requires a hyperlipidemia background, we have bred each of the strains to a common strain (C57BL/6J) that donated transgenes for two dyslipidemia-inducing mutations: human apolipoprotein E-Leiden (APOE-Leiden) and human cholesteryl ester transfer protein (CETP). Genetic differences among the F1 animals arise only from sequence variations present in the individual recipient strains. We term this set of F1 animals the “Ath-HMDP”. Association analysis was used to identify and map phenotypic traits correlated with these known sequence variations. We have analyzed the progeny using a multi-phenotype layered “systems genetics” approach, involving the analysis of molecular as well as clinical phenotypes. The results provide a view of the genetic architecture of atherosclerosis in mice and enable statistical modeling of pathways underlying atherosclerosis. They also serve as a resource for future studies.

## Results

### Strategy for construction and analysis of Ath-HMDP mice

**Fig 1:**
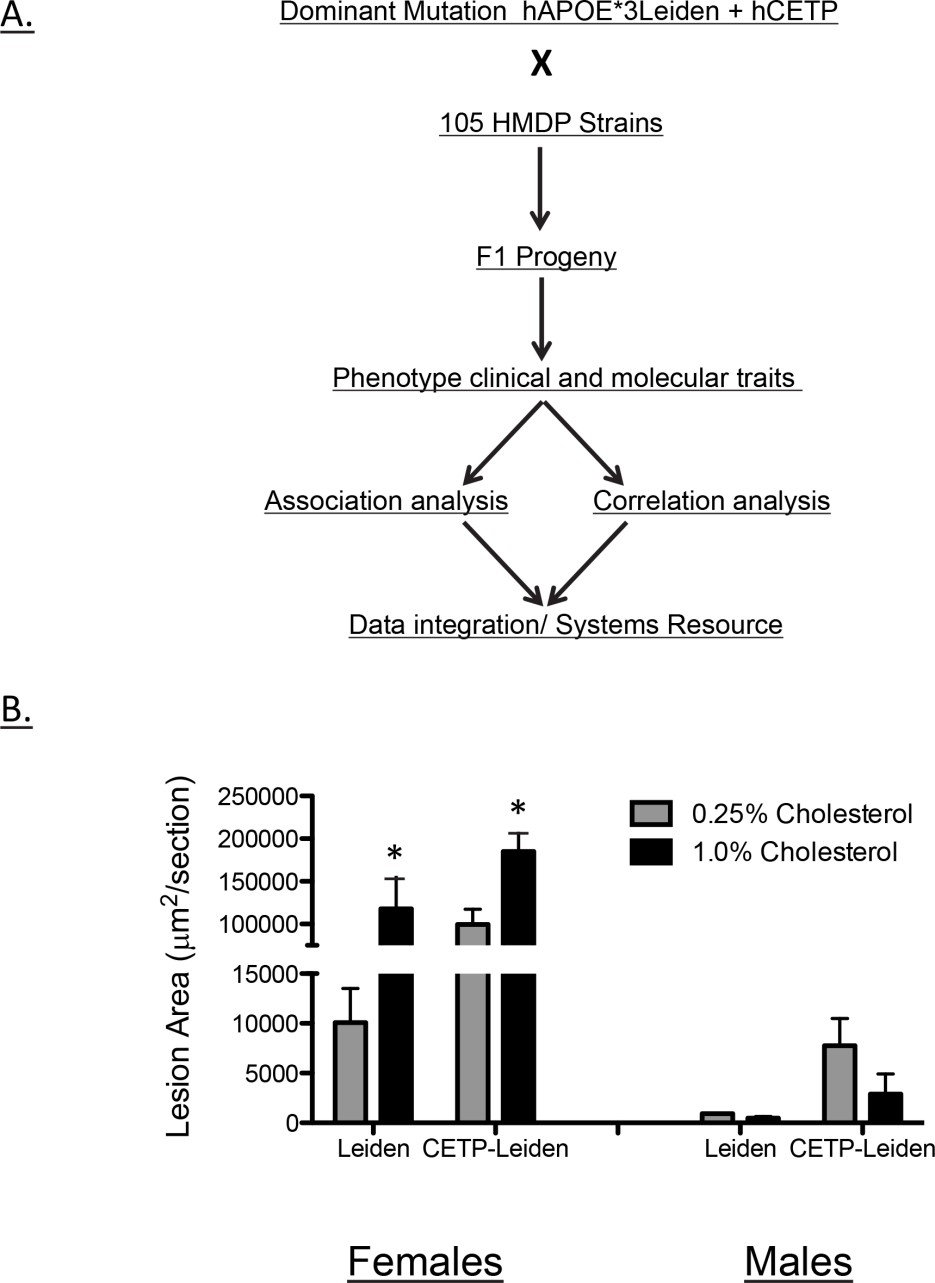
Strategy for construction and analysis of Ath-HMDP mice. (**A**) Flow diagram for construction and analysis of Ath-HDMP mice. Male C57BL/6 mice carrying the dominant human transgenes for ApoE*3 Leiden and CETP were mated with 105 inbred strains from the hybrid mouse diversity panel (HMDP). Beginning at 8 weeks of age, F1 progeny carrying both transgenes were placed on a high-fat diet containing either 1.0% or 0.25% cholesterol for 16 weeks and then measured for a panel of atherosclerosis-related traits. Association and correlative analysis was used to map genetic variation underlying these traits and to identify associated pathways. (**B**) Impact on atherosclerotic lesion area (μm^2^/section) of dietary cholesterol, sex and hAPOE*3Leiden and combined hAPOE*3Leiden/hCETP transgenes on mice (C57BL/6J background) Note scale break at 50,000 μm^2^/section. The diet containing 1.0% cholesterol was associated with a significant increase in atherosclerotic lesion area in females (*, p < 0.05) but not in males.

One difficulty in applying an association strategy to atherosclerosis in a panel of mice is that a sensitizing mutation resulting in hyperlipidemia is required for the development of substantial lesions. The most widely used models are apolipoprotein E null (*Apoe* ^*-/-*^) [18, 19] and LDL receptor null (*Ldlr* ^*-/-*^) mice [20]. Both of these act in a recessive manner with respect to lesion development, and breeding either of these mutations to a homozygous state in numerous strains for an association study is not practical. To circumvent this problem, we developed a strategy in which C57BL/6J mice carrying dominant hyperlipidemia-inducing transgenes were bred to a panel of different strains followed by analysis of atherosclerosis and related traits in the heterozygous (F1) mice (**Fig 1A**). For this purpose, we employed transgenes for human APOE-Leiden and CETP, both of which had been previously used to promote atherosclerosis development in mice in a dominant manner [21, 22]. We found that the combination of both transgenes provided the most robust development of lesions in both female and male mice (**Fig 1B**). We also examined various diets and chose a diet containing 1.0% cholesterol since it significantly enhanced lesion development in females although no significant effect was observed in the smaller lesions observed in male mice (**Fig 1B**).

Our overall experimental design is summarized in **Fig 1A**. We generated mice heterozygous for the APOE-Leiden transgene and homozygous for the CETP transgene on a C57BL/6J background and bred these to each of about 100 HMDP mouse strains with selection of F1 mice expressing both transgenes. Ideally, donor mice homozygous for both transgenes would have been used in these studies, but we found that homozygous APOE-Leiden mice were poor breeders. The F1 mice (which we term “Ath-HMDP”) were maintained on a chow diet until 8 weeks of age and then challenged with a “Western style” high-fat cholesterol containing diet for an additional 16 weeks, at which time they were euthanized and trait phenotypes examined. In addition to atherosclerosis, we examined a variety of metabolic traits that have been associated with atherosclerosis in human populations, including plasma lipid levels, insulin/glucose levels, blood cell levels, obesity and TMAO levels [23]. We then performed high-resolution association mapping and correlation analyses for the traits and integrated the data to identify candidate genes and pathways.

### Plasma lipids, insulin/glucose, and adiposity

We measured levels of plasma lipids in the Ath-HMDP mice on a chow diet at 8 weeks of age as well as after feeding the mice the high fat high cholesterol diet from 8 weeks to 24 weeks of age. Strain-average plasma levels of total cholesterol, LDL/VLDL-cholesterol, HDL-cholesterol and total triglyceride levels varied considerably among the strains (**S1 Fig**). In fact, for individual mice at 24 weeks, HDL-cholesterol levels ranged from undetectable levels to 220 mg/dl, VLDL/LDL-cholesterol levels ranged from 10 to 2,800 mg/dl, and triglyceride levels ranged from 10 to 2,700 mg/dl. As expected, the diet greatly elevated the levels of total- and LDL/VLDL-cholesterol in all the strains, but there remained a strong correlation between values on the chow and Western diets (**S2 Fig**). The correlations of each class of lipids with atherosclerotic lesion area in females are shown in **S3 Fig**. Atherosclerotic lesion size was negatively associated with HDL-cholesterol levels (but only suggestively, p = 0.08) and positively associated with LDL/VLDL-cholesterol levels (p = 0.004) (**Panels A** and **B** in **S3 Fig**). Triglyceride levels were not significantly associated with lesion area (p = 0.41) (**Panel C** in **S3 Fig**). The strengths of associations in male mice are shown in **S1 Table**.

The levels of plasma glucose, insulin (presented in **S4 Fig**) and a measure of insulin resistance (HOMA-IR)[17, 24], were determined. There were large variations in all three parameters, with insulin levels ranging from undetectable levels to over 9,800 pg/ml. Glucose levels ranged from 126 mg/dl to nearly 450 mg/dl, with 75% of the mice having blood glucose levels in excess of 211 mg/dl. Correlation of glucose with atherosclerotic lesion size reached significance (p = 0.016) in males but not in females (p = 0.329) (**Panel E** in **S3 Fig** and **S1 Table**). HOMA-IR, a measure of insulin resistance calculated from glucose and insulin values, ranged between 1.2 and 54. Correlation of HOMA-IR with atherosclerotic lesion size was modest (p = 0.06) in females but not significant in males (p = 0.44) (**Panel F** in **S3 Fig**, **S1 Table**).

The levels of body fat, examined using nuclear magnetic resonance (NMR) spectroscopy, ranged from <10% to nearly 50% adiposity (**S2 Table)**. There was no significant relationship between adiposity and the extent of atherosclerosis among the male mice (r = -0.099, p = 0.381) but a significant inverse relationship among female mice (r = -0.353, p = 0.001) (**S1 Table**). This inverse relationship is consistent with studies in genetically obese (*ob/ob* and *db/db*) mice which have reduced atherosclerosis [25]. This may have to do with increased lipoprotein particle size in obese mice. For example, lipoprotein lipase deficient mice have severe hypertriglyceridemia without increased atherosclerosis [26].

**Fig 2:**
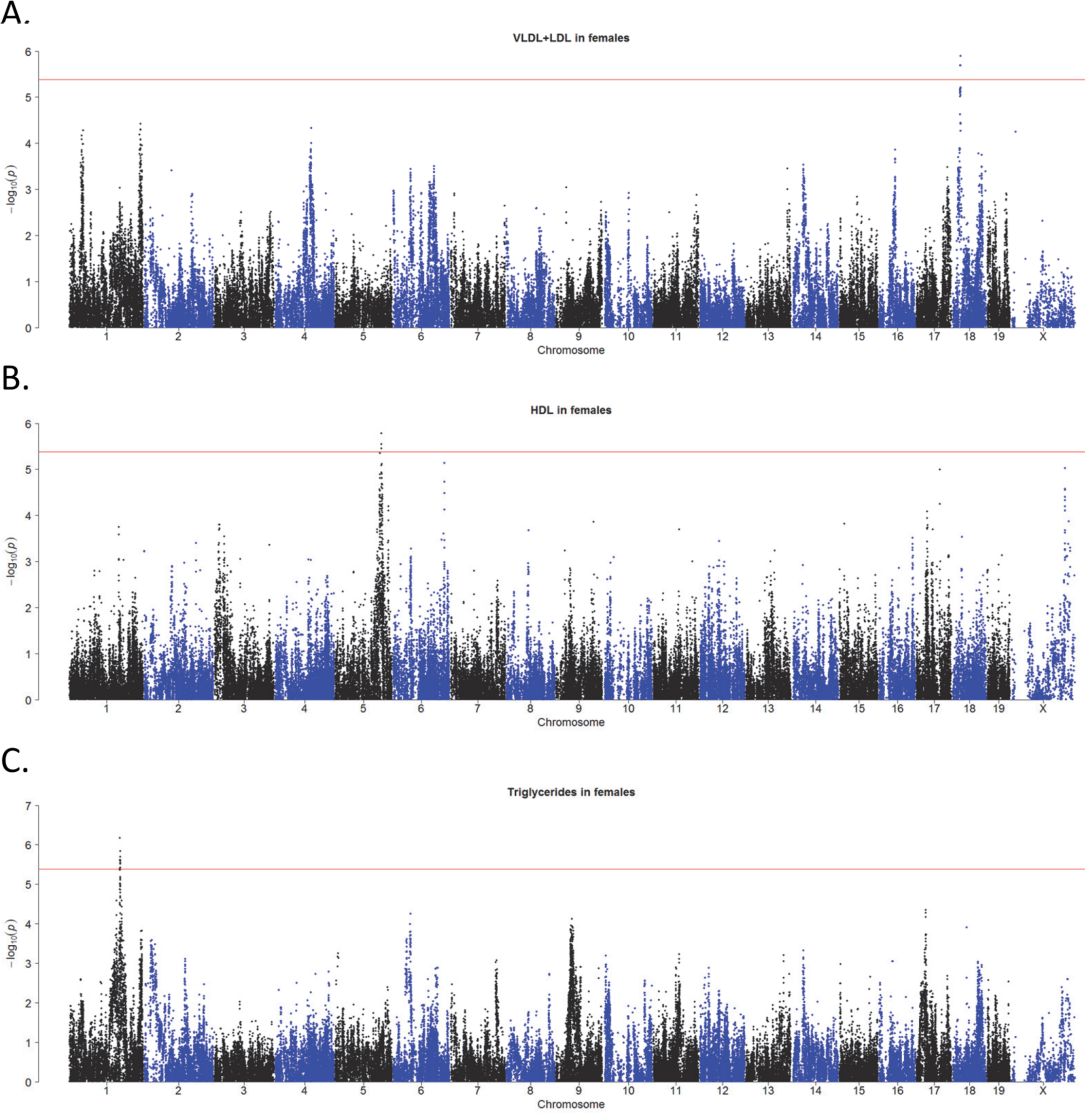
Plasma lipids in the Ath-HMDP. Genome wide association plots for very low-density lipoproteins, VLDL, and low-density lipoproteins, LDL (**A**). High density lipoproteins, HDL (**B**), and Triglycerides (**C**).

**Fig 3:**
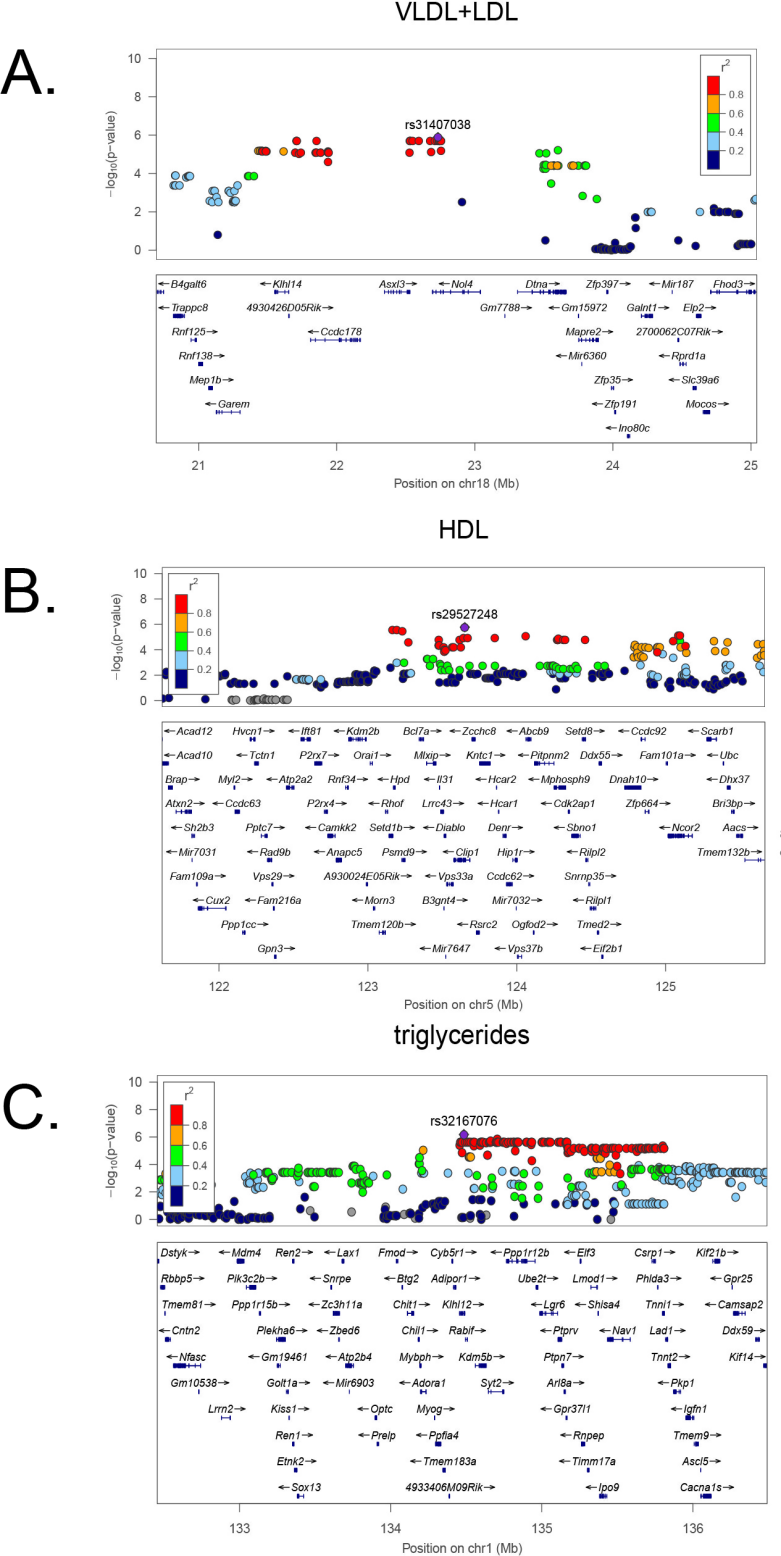
High resolution regional plots of plasma lipid loci in Ath-HMDP. Locus Zoom plots [118] are shown for the VLDL+LDL locus on Chr 18 (**A**), the HDL locus on Chr 5 (**B**) and the triglycerides locus on Chr 1 (**C**). Physical locations of genes are denoted by blue horizontal bars. The linkage disequilibrium (LD) of the SNPs with the lead SNP at the locus is denoted by the color of the SNP. Red indicates SNPs that are highly correlated (in strong LD with each other) and defines the critical region for candidate gene selection.

Using association analysis with correction for population structure, we mapped major loci contributing to lipoprotein levels in the Ath-HMDP. The regions identified as most likely to contain the causal gene (or genes) were those in strong linkage disequilibrium with the peak SNP (determined by calculated r^2^ SNP correlations greater than 0.8) and exceeding the FDR cutoff of 5%, corresponding to the association p-value of 1.3×10^−5^ [27]**.** A list of all significant loci (FDR < 5%) is provided in **S3 Table**, but we highlight several of the associations for plasma lipids identified in females. A significant association was identified for VLDL/DL cholesterol on Chr 18 at 21 Mb (**Figs 2A and 3A**), HDL cholesterol on Chr 5 at ~120 Mb (**Figs 2B and 3B**), and triglycerides on Chr 1 at ~135 Mb (**Figs 2C and 3C)**. The QTL for triglycerides is novel, while the Chr 18 peak has previously been associated with HDL cholesterol but not VLDL/LDL or total cholesterol [28] and the Chr 5 peak is within the QTL boundaries of 2 previously reported HDL QTL, *Hdlq8* and *Chldq12* **[28, 29]**. Some loci observed on chow diets are obscured on the hyperlipidemia background; for example, on a chow diet, the *Apoa2* locus on Chr 1 is a major determinant of HDL-cholesterol levels [12] suggesting that gene x diet interactions influence disease susceptibility.

### Atherosclerosis: Aortic lesion area

**Fig 4:**
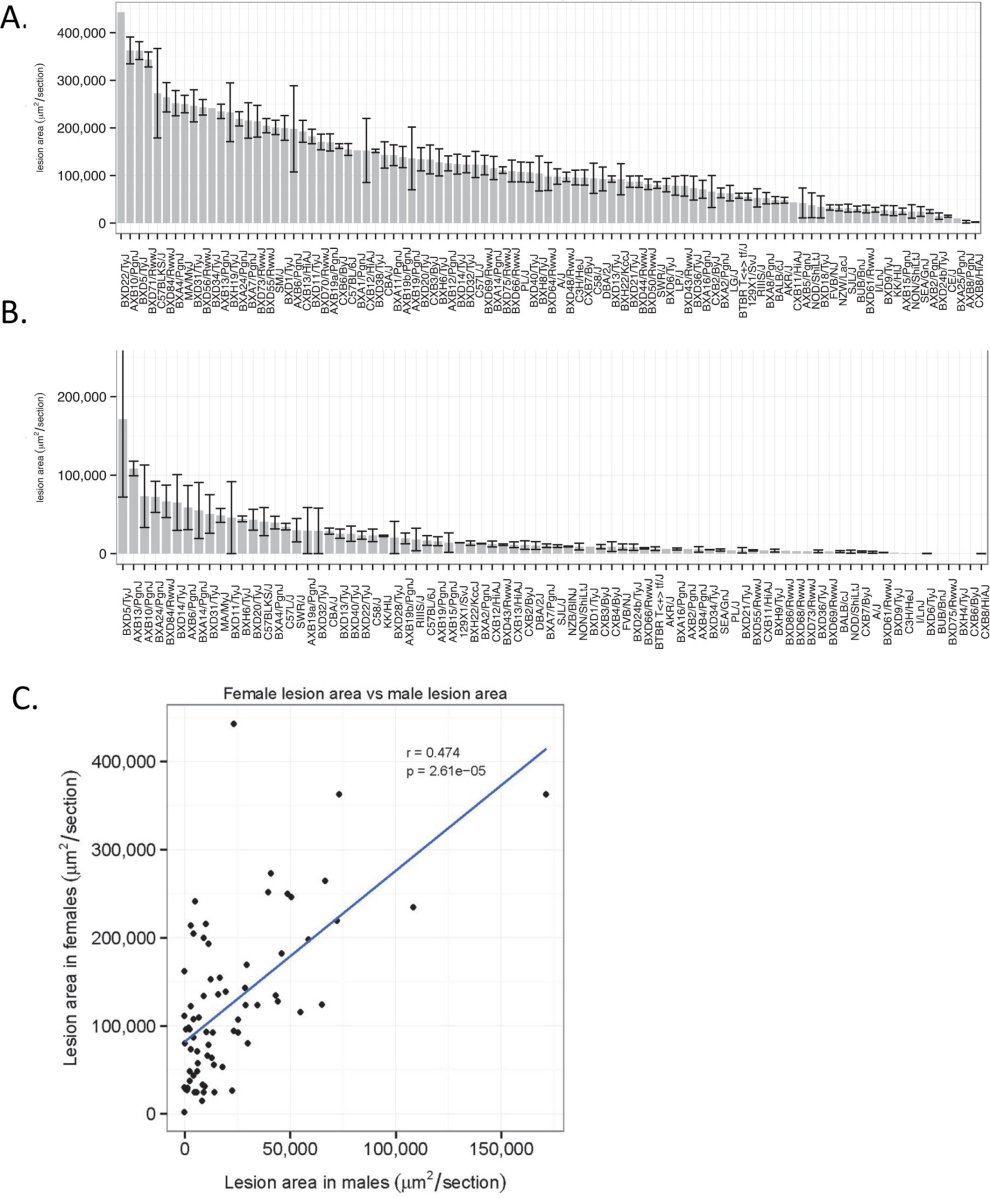
Atherosclerosis in Ath-HMDP mice. Atherosclerotic lesion size (μm^2^ ± SEM) in the proximal aorta and aortic sinus were quantitated for 697 female mice (**A**) and 281 male mice (**B**) using oil red—O staining. In each panel, strains are arranged in rank order by strain-average lesion area. (**C**) Correlation between strain-average lesion areas in male and female Ath-HMDP mice.

**Fig 4**shows the distribution of atherosclerotic lesion areas in the proximal aorta in the Ath-HMDP mice. Strain-average lesion areas in females ranged as high as 400,000 μm^2^ in several recombinant inbred (RI) strains to less than 2,500 μm^2^ for resistant strains (**Fig 4A**). Strain-average lesion areas in males tended to be much lower but also varied widely, from about 170,000 μm^2^ to negligible (**Fig 4B**). Strain C57BL/6J mice, with lesion sizes of about 150,000 μm^2^/section for females and 19,000 μm^2^/section for males, were approximately intermediate in the panel of strains. The sizes of lesions correlated significantly between males and females (r = 0.47, p = 2.61 X 10^−5^) (**Fig 4C**) but, clearly, the ratio of lesion sizes in the sexes varied widely, indicating the existence of gene-by-sex interactions. The range of lesion-areas is consistent with previous results from a small number of strains on either *Apoe* ^*-/-*^ or *Ldlr* ^*-/-*^ backgrounds as well as genetic crosses [30, 31]. We were also interested in the site specific regulation of atherosclerosis and dissected the brachiocephalic arteries from the first 300 mice, but did not observe significant lesion development. We thus focused our efforts on the aortic root.

**Fig 5:**
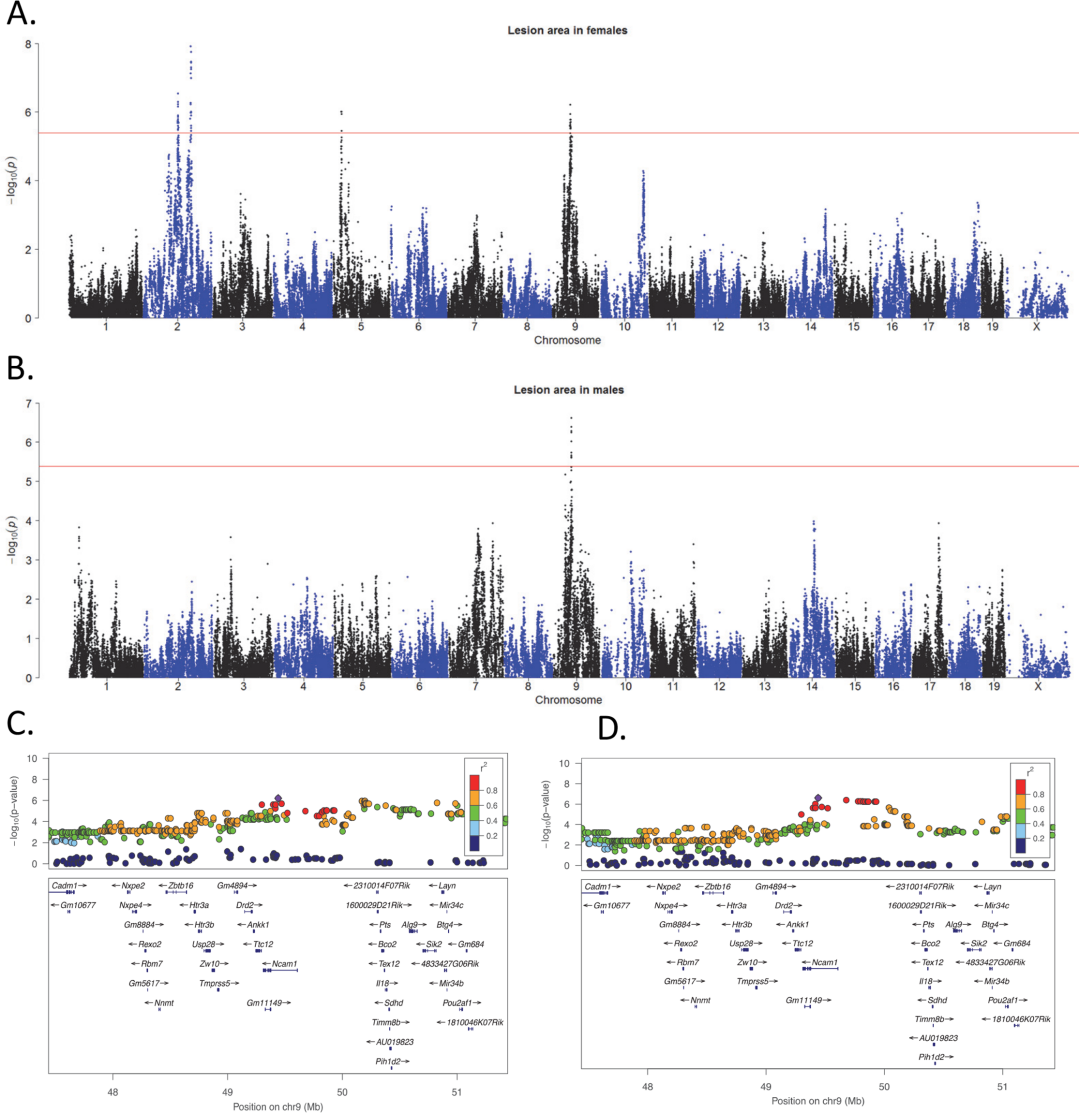
Genetic regulation of atherosclerotic lesion area in HMDP mice. Loci detected for aortic sinus lesion area are shown for (**A**) female mice, (**B**) male mice. The X-axis shows genomic position while the Y-axis indicates −log10 of the p-value following correction for population structure, as described in Methods. The horizontal red line indicates the HMDP cutoff for genome-wide significance; p = 4.2 X 10^−6^. [12] (**C, D**) LocusZoom plots [118] of the genetic association results are shown for (**C**) female mice and (**D**) male mice. Physical locations of genes are denoted by blue horizontal bars. The linkage disequilibrium (LD) of the SNPs with the lead SNP at the locus is denoted by the color of the SNP. Red indicates SNPs that are highly correlated (in strong LD with each other) and defines the critical region for candidate gene selection.

We next sought to determine the loci contributing to lesion development. Using an FDR cutoff of 5%, corresponding to the association p-value of 1.3×10^−5^ [27], we were able to identify 4 genome-wide significant loci in females and 1 locus in males significantly associated with atherosclerotic lesion size (**Fig 5A and 5B**). The locus on Chr 9, encompassing about 1 Mb between 46 and 47 Mb of the chromosome, was observed in both sexes (**Fig 5C and 5D**) and corresponds to a locus previously identified in genetic crosses between strains C57BL/6J and C3H/HeJ [32]. High-resolution regional plots of the other 3 loci in females are presented in **S5 Fig**. The two loci on Chr 2 are clearly distinct because conditioning on one does not affect the association for the other (**S6 Fig**). Due to differences in breeding, the number of F1 mice differed considerably between strains (**S2 Table**). When we performed association analysis using only strains with 3 or more mice, the significant regions exhibited similar p-values but a suggestive peak on Chr 10 became significant (**S7 Fig**).

### Blood cell levels: Association of monocytes with atherosclerosis

Recent studies have highlighted the relationship between blood cells and cardiovascular disease[33–35]. We quantitated blood cell levels and correlated these with the extent of lesion development. The number of granulocytes, monocytes and percent granulocytes were positively correlated with lesions but did not reach statistical significance (**S4 Table)**. The percentage of white cells that were monocytes was significantly and positively correlated with atherosclerosis (r = 0.231, p = 0.023) (**S4 Table**)**.**

We mapped major loci contributing to the blood cell levels (**S3 Table**), and identified multiple loci affecting these traits. We detected an association for the percentage of white blood cells that were monocytes on Chr 8 at 36 Mb and total monocyte counts were regulated by loci on Chr 9 and 13 (**S3 Table**).

### Plasma cytokine levels: Association of KC, the mouse homolog of IL-8, with atherosclerosis

**Fig 6:**
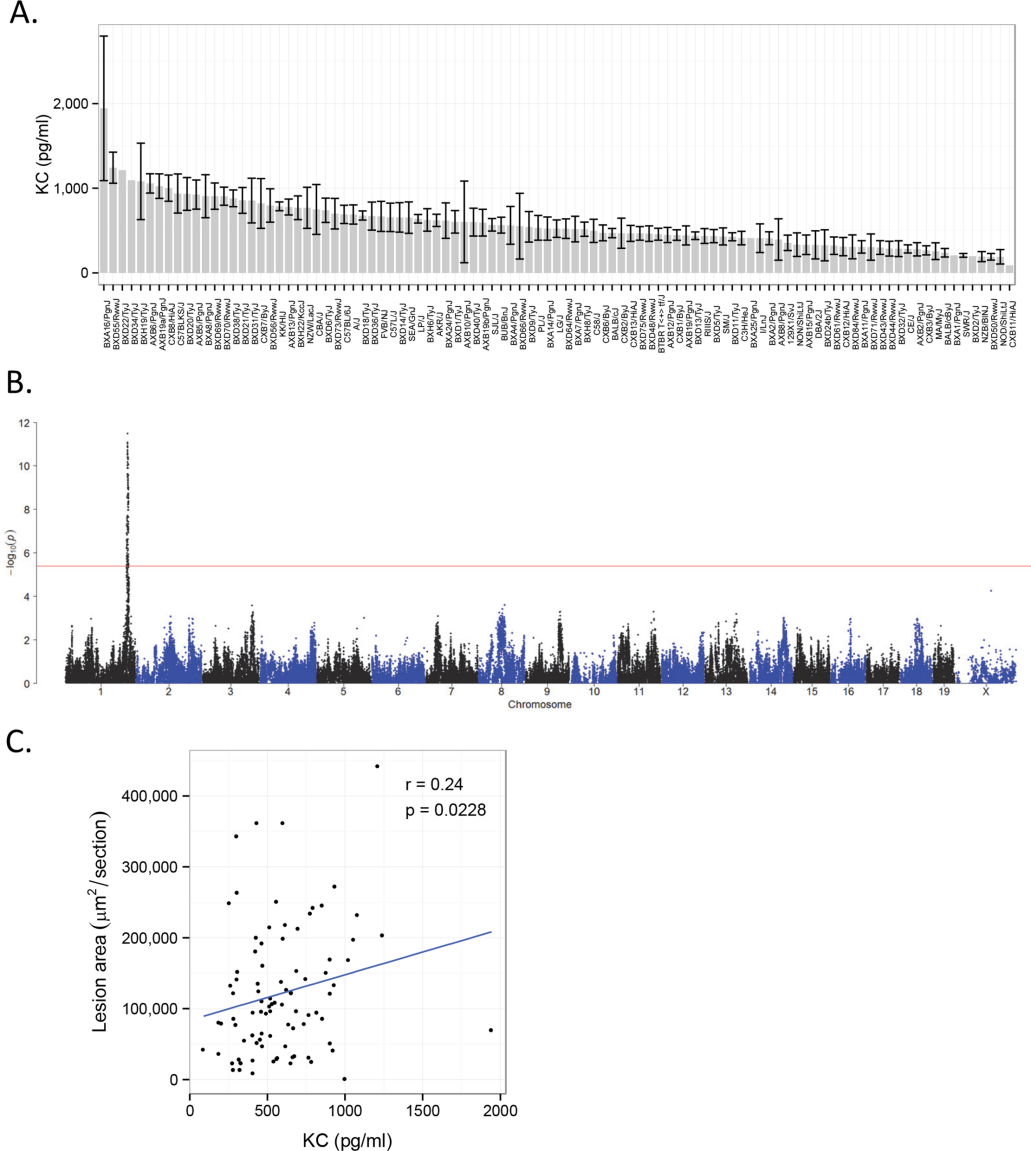
Circulating KC levels are under genetic regulation and correlate with atherosclerosis in the Ath-HMDP. Plasma levels of KC (keratinocyte-derived chemokine)(pg/ml), a homolog of human Il-8 were quantitated for 676 female mice. Strain-average levels of KC (± SEM) are arranged by decreasing rank order (**A**). Association mapping identified a single strong locus on Chr 1 for plasma KC (**B**). X-axis is genomic position and Y-axis is −log10 of the p-value for association following correction for population structure, as described in Methods. The horizontal red line indicates the HMDP cutoff for genome-wide significance; p = 4.2 X 10^−6^. [12] Correlation between atherosclerosis and plasma KC levels (**C**). Individual points indicate strain averages for atherosclerotic lesion area (μm^2^/section) and KC (pg/ml).

Cytokines are clearly important in atherosclerosis as indicated by transgenic studies in mice as well as human GWAS studies [4]. Although cytokines usually act in a local manner, we sought to examine circulating levels as a possible indicator of cytokine expression, using an immune capture microbead system. A list of the cytokines that were quantitated (including their full names and abbreviations) is presented in **S5 Table**. Those that could be accurately quantitated in a large fraction of strains included KC, G-CSF, IL-10, MCP-1, MIG, MIP-1a, and MIP-1b. All varied widely among the strains and their levels are shown in **Fig 6A** and **S8 Fig**. Of these, only KC exhibited a significant correlation with atherosclerosis (r = 0.24, p = 0.023) (**Fig 6C**). KC levels mapped to a locus on Chr 1 (**Fig 6B**) that has been previously shown to be associated with multiple cardiovascular risk factors [3].

### Macrophage lipid loading: Lack of association with atherosclerosis

**Fig 7:**
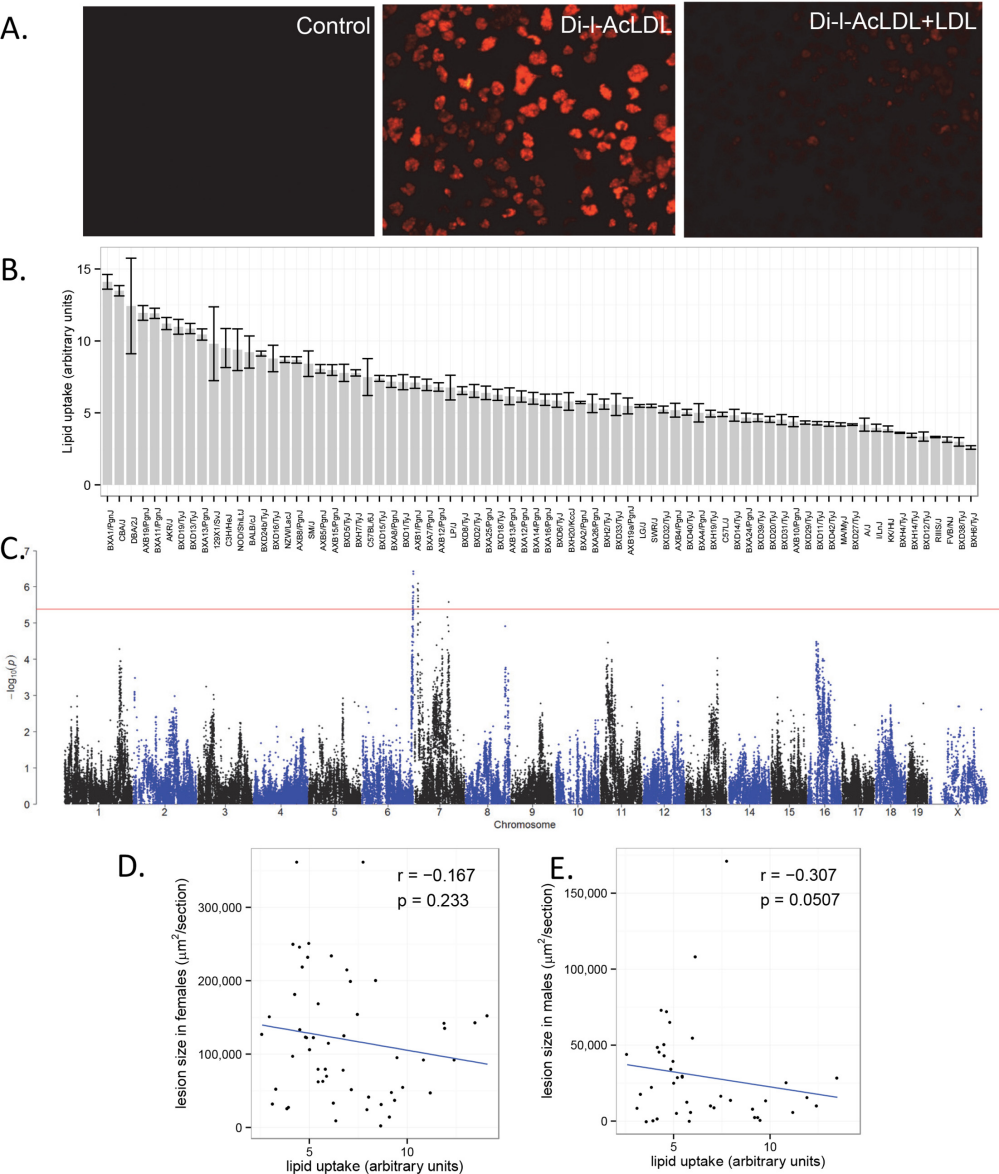
Uptake of DiI-AcLDL by peritoneal macrophages in HMDP mice. (**A**) Representative fluorescence levels following culture of peritoneal macrophages in control cultures and cultures labeled with DiI-AcLDL in the absence or presence AcLDL competition. Levels of DiI-AcLDL uptake measured by relative fluorescence per cell, were quantitated for males of 74 strains. Strains are arranged in decreasing rank order by strain-average DiI fluorescence. (**B**). Association mapping identified loci on Chromosomes 6 and 7 for DiI-AcLDL uptake. The horizontal red line indicates the HMDP cutoff for genome-wide significance; p = 4.2 X 10^−6^. [12] (**C**). X-axis is genomic position and Y-axis is −log10 of the p-value following correction for population structure, as described in Methods. Correlation of atherosclerosis in females **(D)** and males **(E)** with *in-vitro* lipid uptake in macrophages. Individual points indicate strain averages for atherosclerotic lesion area (μm^2^/section) and DiI-AcLDL uptake (relative fluorescence).

The uptake of modified LDL and VLDL particles by macrophages to produce cholesterol-loaded foam cells is a key aspect of atherosclerosis. To examine whether genetic differences in macrophage loading might contribute to lesion development, we quantitated the uptake of acetylated LDL (AcLDL) by peritoneal macrophages from the HMDP mice. Peritoneal macrophages appear to be a suitable surrogate since they show expression of surface markers typically found in atherosclerotic lesion macrophages such as SR-A, CD36, CD68, F4/80, MOMA-2, Ly6C, and CD11b [36–40]. Macrophages were incubated with DiI-AcLDL for 4 hours in 96-well microtiter plates at a density of 3×10^5^ cells/well, washed, and the fluorescence level quantitated using a plate reader. In our initial studies, we observed reproducible differences in the uptake of AcLDL labeled with the fluorescent dye DiI (Panels A and B in **S9 Fig**). Moreover, we observed that uptake was relatively linear over a 24-hour period (Panel C in **S9 Fig**). Representative cultures are shown in **Fig 7A**. The uptake varied greatly among the strains over an approximately 5-fold range (**Fig 7B**), and we identified two significant loci controlling the loading of macrophages by AcLDL, on Chr 6 at 149 Mb, rs30709278 (p = 3.82 x10^-7^) and on Chr 7 at 13 Mb, rs37775929 (p = 8.22 x10^-7^) (**Fig 7C**). These loci do not contain the candidate genes *Cd36* or *Msr1*, previously implicated in cholesterol loading of macrophages, nor did we observe significant correlation between the mRNA levels of *Cd36* and *Msr1* with AcLDL loading [41]. Moreover, the uptake of AcLDL was not correlated with atherosclerosis (**Fig 7D**), suggesting that AcLDL is not a good surrogate for the modified LDL found in lesions, or that cholesterol loading is not a limiting process in lesion development.

### Atherosclerosis: Aortic lesion composition

**Fig 8:**
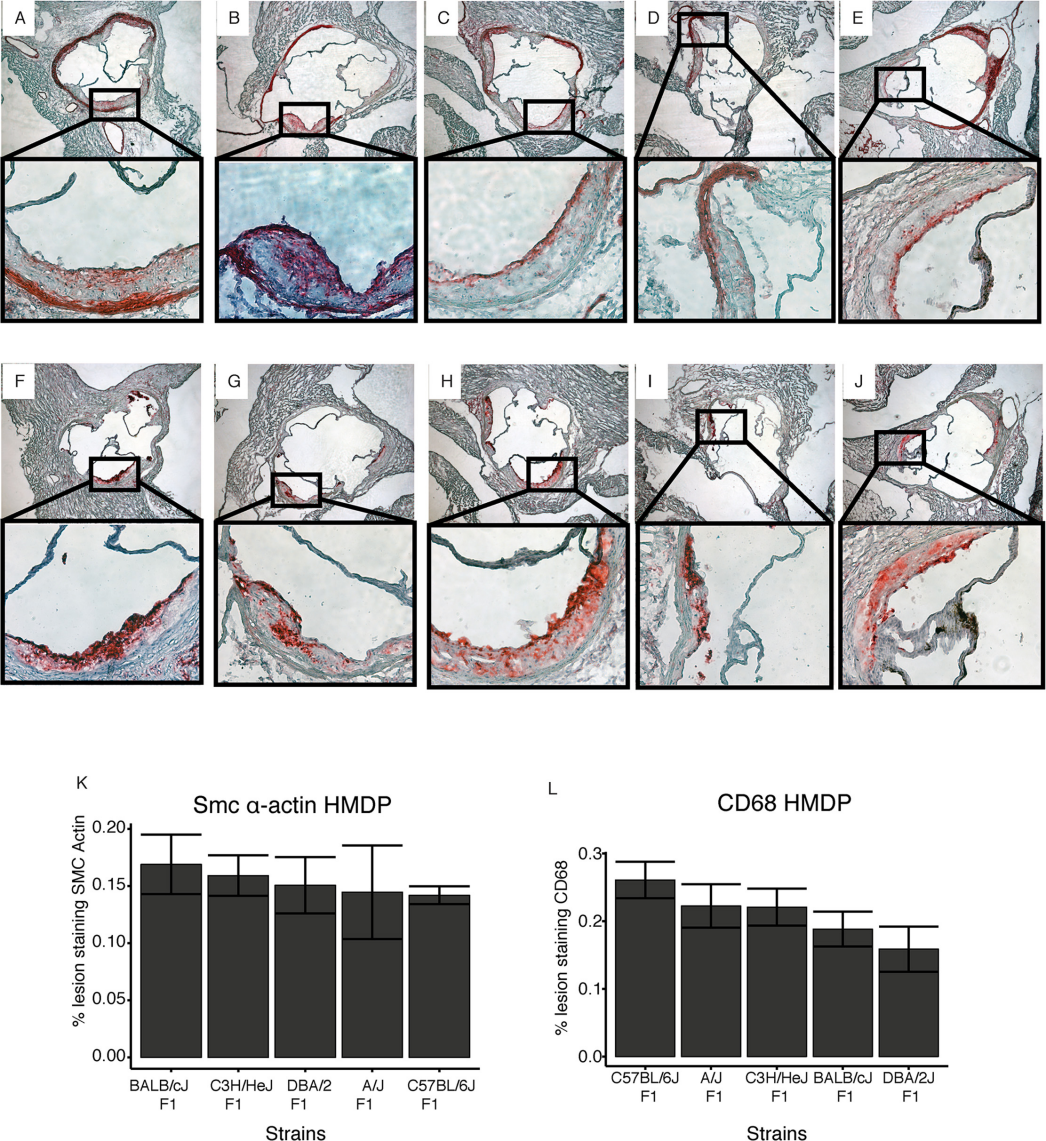
Lesion morphology in 5 progenitor inbred strains. CD68+ cells but not smooth muscle actin cells differ among strains comprising the HMDP. Representative results from immunostained lesions for sm-α actin are shown in Panels **A-E** and CD68 in panels. C57BL/6J (**A,F**), A/J (**B,G)**, BALB/cJ (**C, H**), C3H/HeJ (**D, I**), DBA/2J (**E, J**). (**K, L**) Impact of genetic background on lesion morphology in C57BL/6, A/J, C3H/HeJ, BALB/c and DBA/2, 5 progenitor strains for the HMDP recombinant inbred strains. Immunohistological staining for macrophages (CD68) (**K**) or smooth muscle cells (smooth muscle α-actin) (**L**) was measured as percent of total atherosclerotic lesion area ± SEM.

To determine whether genetic factors contributed to lesion morphology as well as lesion size, we examined lesions with antibodies specific for macrophages (CD68) or smooth muscle cells (SM-α actin). We chose to examine the five progenitors of the four recombinant inbred strain-sets that comprise the HMDP (C57BL/6J, DBA/2J, C3H/HeJ, A/J, and BALB/cJ) since they provide much of the power for the association analyses. Representative sections are shown in **Fig 8A–8J**. Three histological sections from each of 5 female mice were quantitated for fraction of lesion area that shows staining for SM-α actin or CD68 and the results are plotted in **Fig 8K and 8L**, respectively. There were significant differences in CD68 staining, presumably indicating alterations in inflammatory pathways. We did not detect significant differences in the percentage of lesions staining positive for alpha actin staining. Genetic contributions to lesion morphology have been noted in some previous studies in mice [42–44].

### Plasma metabolite levels: Associations of TMAO and arginine metabolites with atherosclerosis

**Fig 9:**
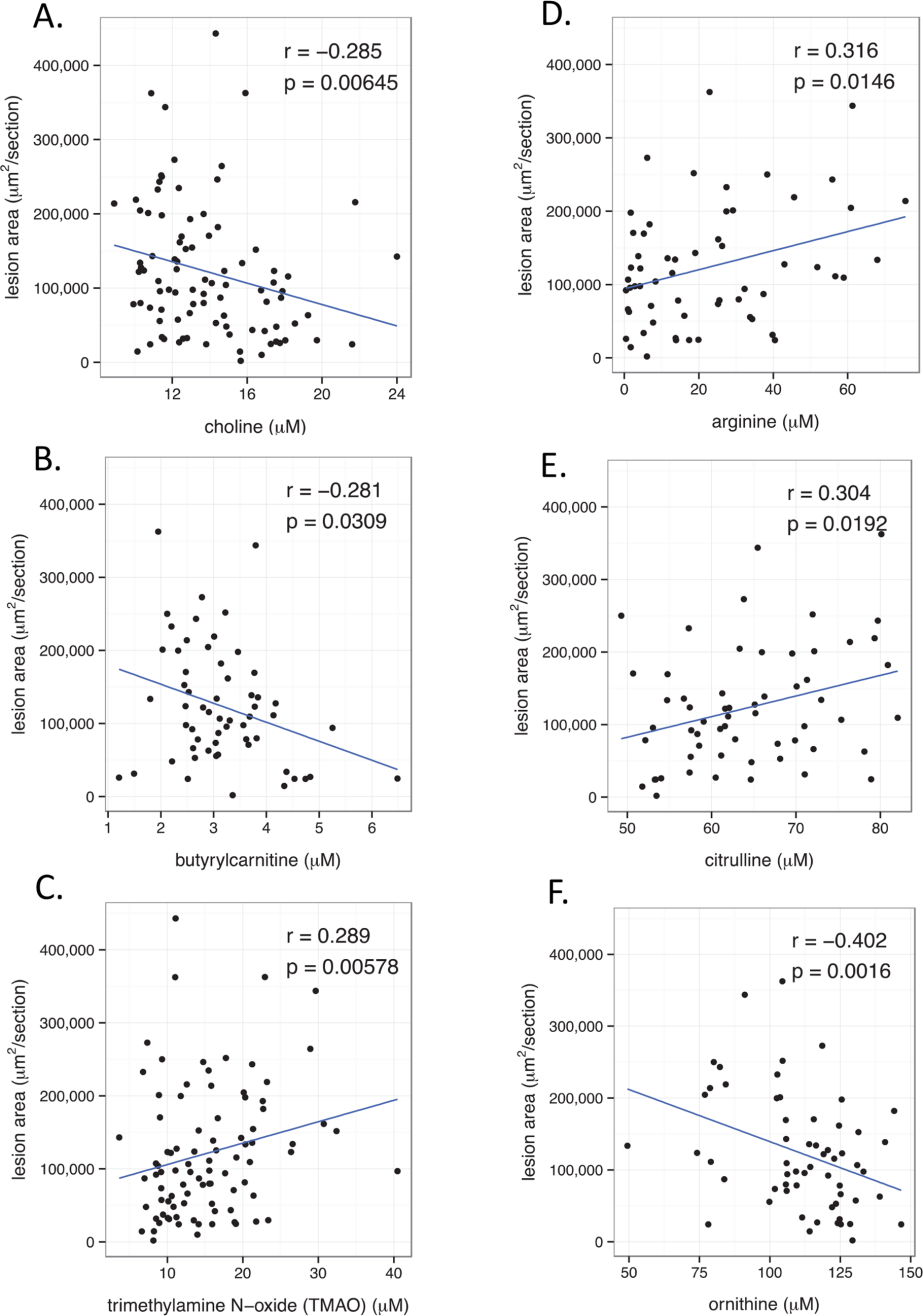
Relationships of plasma polar metabolite concentrations with atherosclerosis in Ath-HMDP. Correlations of atherosclerosis with plasma concentrations of choline (r = -0.285, p = 0.00645) (**A**), butyryl-carnitine (r = -0.281, p = 0.0309) (**B**), TMAO (r = 0.289, p = 0.00578) (**C**), arginine (r = 0.316, p = 0.0146) (**D**), citrulline (r = 0.304, p = 0.0192) (**E**), and ornithine (r = -0.402, p = 0.0016) (**F**). Individual points indicate strain averages for atherosclerotic lesion areas (μm^2^/section) and metabolite concentrations (μM).

We quantitated the levels of 31 polar metabolites in plasma of a subset of female strains. Of these, butyryl-carnitine, choline, TMAO, arginine, citrulline and ornithine exhibited significant correlations with atherosclerosis, and their strain average levels and correlations with atherosclerosis are presented in **S10 Fig and Fig 9**. Most notably, the recently discovered risk factor for atherosclerosis, TMAO [23], was positively correlated with atherosclerosis, r = 0.29, p = 0.006 (**Fig 9C**). On the other hand, the levels of choline, a metabolic precursor of TMAO [45], were negatively correlated with atherosclerosis (**Fig 9A**).

The inter-related metabolites arginine, ornithine and citrulline are involved in the formation of nitric oxide (NO), an important regulator of vascular tone, blood flow and pressure [46–48]. Ornithine, which was negatively correlated with lesion area (r = -0.40, p = 0.002) (**Fig 9F**), is a breakdown product of arginine via the enzyme arginase I. Levels of arginine were positively correlated with lesion area (r = 0.32, p = 0.015) (**Fig 9D**) as were levels of citrulline (r = 0.30,p = 0.02) (**Fig 9E**). As discussed below, we quantitated global gene expression in livers of female mice from 96 strains in the Ath-HMDP using Affymetrix HT-MG 430 PM microarrays. Several genes known to regulate these metabolites were highly associated with plasma concentrations. For example, hepatic expression of *Arg1*, encoding arginase 1, was highly negatively correlated with ornithine (r = -0.45, p = 0.0003) and positively correlated with arginine levels (r = 0.323, p = 0.013). This suggests either that the ornithine and arginine in plasma are not of hepatic origin or that there is feedback regulation of *Arg1*. It is noteworthy that the levels of arginase I in macrophages are a marker of M2 (anti-inflammatory) as compared to M1 (pro-inflammatory) macrophages [49, 50].

We performed association analyses to identify loci regulating circulating metabolites associated with atherosclerosis. Notably we identified loci for TMAO levels on Chr 10 at 98 Mb, (p = 6×10^−6^) and on Chr 16 at 94 Mb (p = 1×10^−6^) (**S3 Table**). Arginine was associated with a major locus on Chr 7 at 44Mb, rs32257964 (p = 2×10^−8^) but also exhibited complex regulation with additional loci on Chrs 1, 4, 11 and 13 (**S3 Table**). There are 13 hepatic genes with eQTL that are in strong LD with the associated SNP at the Chr 7 locus. Of these, *Nosip* is an interesting candidate as it has a strong *cis*-eQTL (p = 8.7×10^−22^) and overexpression of *Nosip* has previously been shown to reduce NO synthesis *in-vitro* [51].

### Gene expression in aorta and liver: Gene network modeling

To help identify pathways underlying atherosclerosis, we quantitated global gene expression in livers and aortas of three individual female mice for each strain using Affymetrix HT-MG 430 PM microarrays. For this analysis, we used only strains where 3 or more female mice were available (104 strains for aortas and 96 strains for livers).

**S6 Table** lists genes whose expression is most strongly positively or negatively correlated with atherosclerotic lesion size. These genes were analyzed using DAVID to test for enrichment of Gene Ontology (GO) categories. Overall there were broad differences in the function of genes correlated with atherosclerosis in the liver and the aorta. The genes most highly correlated with atherosclerosis were enriched for immune response genes in the liver and for defects in smooth muscle cell function and structure in the aorta (**Table 1**).

**Table 1:** GO categories enriched for atherosclerosis susceptibility using expression data from aorta and liver.

Tissue	Category	Term	Count	%	p-Value	List Total	Pop Hits	Pop Total	Fold Enrichment	Bonferroni	Benjamini
Aorta	KEGG PATHWAY	mmu05414:Dilated cardiomyopathy	19	2.35	2.28x10^-07^	226	89	4524	4.273	3.46 x10^-05^	1.73 X10^-05^
Aorta	GOTerRM_CC_FAT	GO:0015629~actin cytoskeleton	29	3.59	2.44 x10^-07^	500	199	10451	3.0460	9.51 x10^-05^	9.51 X10^-05^
Aorta	SP_PIR_KEYWORDS	actin-binding	30	3.71	4.41 x10^-07^	720	221	15456	2.9140	1.72 x10^-04^	1.72 x10^-04^
Aorta	GOTERM_MF_FAT	GO:0008092~cytoskeletal protein binding	44	5.45	3.47 x10^-07^	518	405	11085	2.3248	2.43 x10^-04^	2.43 x10-04
Aorta	SP_PIR_KEYWORDS	muscle protein	13	1.61	1.77 x10^-06^	720	49	15456	5.69	6.89 x10^-04^	3.45 x10^-04^
Aorta	GOTERM_MF_FAT	GO:0003779~actin binding	33	4.08	3.04 x10^-06^	518	283	11085	2.495	0.00213	0.001
Aorta	GOTERM_CC_FAT	GO:0044449~contractile fiber part	16	1.98	8.71 x10^-06^	500	84	10451	3.981	0.00338	0.0016
Aorta	GOTERM_CC_FAT	GO:0043292~contractile fiber	16	1.98	2.71 x10^-05^	500	92	10451	3.635	0.01047	0.0035
Aorta	GOTERM_BP_FAT	GO:0030036~actin cytoskeleton organization	23	2.84	5.88 x10^-06^	522	164	11331	3.044	0.01357	0.0045
Liver	GOTERM_BP_FAT	GO:0006955~immune response	47	5.85	1.44 x10^-09^	479	414	11331	2.68	3.05E-06	3.05E-06
Liver	INTERPRO	IPR006117:2-5-oligoadenylate synthetase, conserved site	6	0.74	3.38 x10^-06^	672	7	15054	19.201	0.00376	0.0018
Liver	PIR_SUPERFAMILY	PIRSF001990:class I histocompatibility antigen	7	0.87	1.14 x10^-05^	305	13	6642	11.72	0.00385	0.0038
Liver	SP_PIR_KEYWORDS	immune response	21	2.61	3.58 x10^-05^	669	167	15456	2.9051	0.01377	0.013
Liver	SP_PIR_KEYWORDS	immune response	21	2.61	3.58 x10^-05^	669	167	15456	2.905	0.0137	0.013
Liver	INTERPRO	IPR001039:MHC class I, alpha chain, alpha1 and alpha2	7	0.87	1.74 x10^-04^	672	20	15054	7.840	0.176	0.038
Liver	INTERPRO	IPR018952:2'-5'-oligoadenylate synthetase 1, domain 2/C-terminal	6	0.74	1.06 x10^-04^	672	12	15054	11.200	0.1113	0.038
Liver	INTERPRO	IPR004020:Pyrin	7	0.87	2.34 x10^-04^	672	21	15054	7.467	0.230	0.042
Liver	INTERPRO	IPR006116:2-5-oligoadenylate synthetase, ubiquitin-like region	6	0.741	1.65 x10^-04^	672	13	15054	10.3392	0.168	0.045

**Fig 10:**
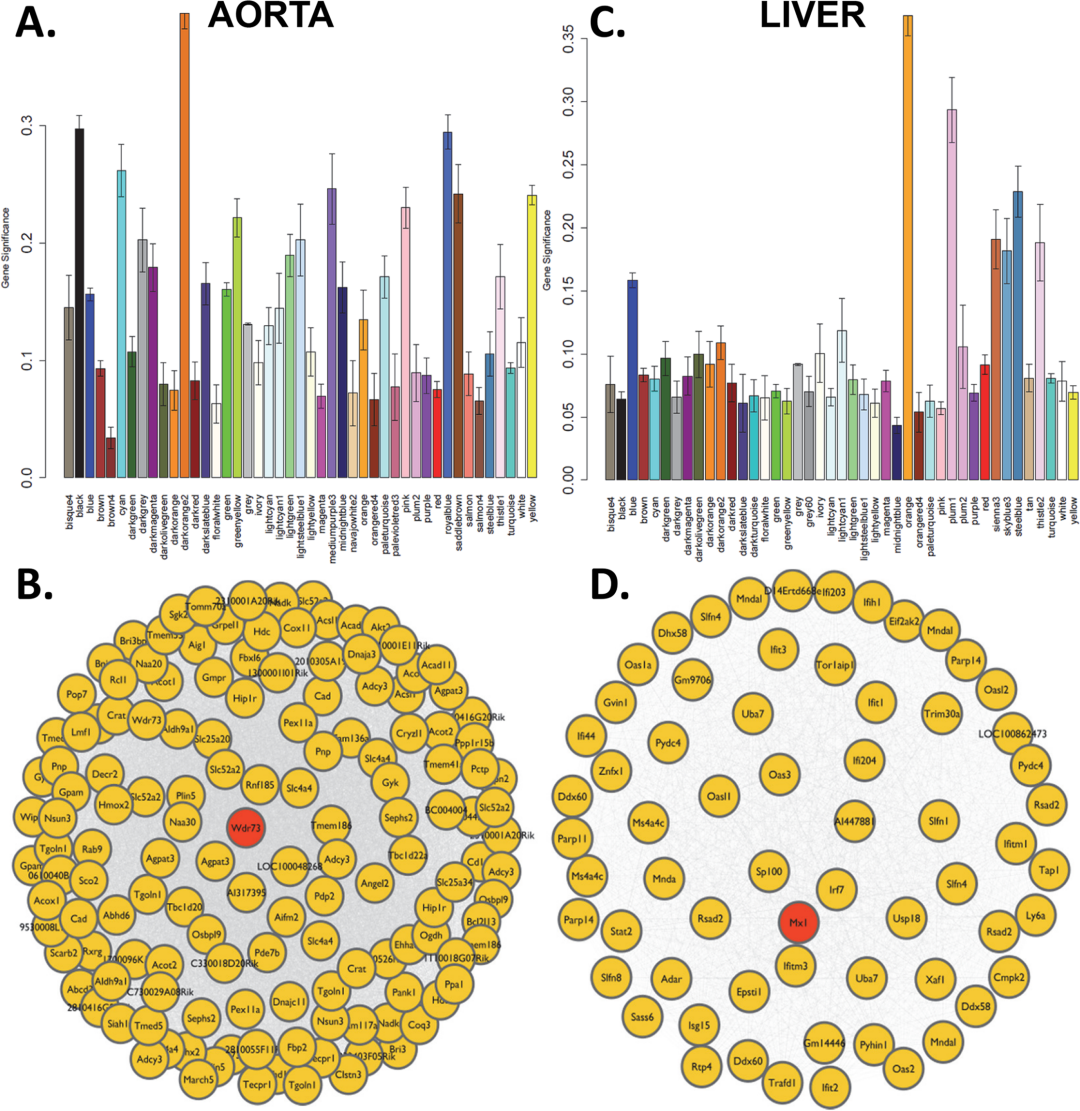
Network analysis of hepatic and aortic gene expression. Gene coexpression networks were generated using Weighted Gene Coexpression Network Analysis (WGCNA) applied to aorta and liver gene expression profiles. The networks were partitioned into modules based on their topological overlap and the correlation of each module with lesion size was determined by calculating the average significance for all genes in the module. (**(A)** aorta and **(C)** liver). The most significantly correlated module were darkorange2 (**B**) for aorta and orange (**D**) for The most connected gene for each module (“hub gene”, shown in pink) was Wdr73 for aorta and Mx1 for liver.

We also analyzed the expression data using the weighted gene co-expression network analysis (WGCNA) [52] to model co-expression networks in the aorta and liver and to understand the association of gene networks with lesion size. WGCNA is a global analysis aimed at identifying genetic pathways associated with clinical traits, in this case atherosclerosis, and is used to aggregate gene expression into groups of highly co-expressed genes, called modules. The first principle component of each module was then related to atherosclerosis to identify gene clusters associated with the disease. We identified 43 and 45 co-expressed gene modules, ranging in size from 15 to 794 genes, in the aorta and liver, respectively. The most significantly correlated aortic module (darkorange2, r = 0.46, correlation p = 4.3×10^−5^) contained 155 genes (**Fig 10A)**. This module was significantly enriched for the Gene Ontology “acyl-CoA metabolic process” category (enrichment p = 2.6×10^−6^). Among the genes in the module, *Wdr73* was a hub, exhibiting the strongest connections with all the other genes (**Fig 10B)**. *Wdr73* has been implicated in a human GWAS of periodontitis [53] and in Galloway-Mowat syndrome, a rare autosomal-recessive condition characterized by nephrotic syndrome associated with microcephaly and neurological impairment [54]. However, the function of this gene is not known and its role in atherosclerosis has not been studied. In the liver, the most significantly correlated module (orange, r = 0.46, correlation p = 3.5×10^−6^) contained 56 genes (**Fig 10C)** and was highly enriched for the Gene Ontology “defense response to virus” category (enrichment p = 5.2×10^−23^). The hub gene of the module was *Mx1* which encodes a guanosine triphosphate (GTP)-metabolizing protein that is induced by type I and type II interferons (**Fig 10D)**. The module also contained several inflammatory genes, such as *Irf7* (interferon regulatory factor 7), *Ifit2* and *Ifit3* (interferon-induced protein with tetratricopeptide repeats 2 and 3).

### Expression quantitative trait loci (eQTL)

To model causal interactions, help prioritize candidate genes at GWAS loci and examine gene-by-gene interactions, we mapped loci controlling gene expression traits (eQTL) in aorta and liver. We utilized the FaST-LMM algorithm to perform association while correcting for population structure [55]. Approximately 200,000 SNPs that segregate among the HMDP strains were chosen based on minor allele frequency greater than 10% [56]. Loci in which peak SNPs mapped within 2Mb of the gene whose expression was regulated were considered “local” SNPs while SNPs mapping elsewhere were considered “distal” and presumably trans-acting eQTL. We calculated the significant p-value cutoff for local and distal associations separately for both tissues. In aorta, we identified a total of 3,718 local eQTLs and 5,837 distant eQTLs at p-values of 8.4×10^−4^ and 1.5×10^−6^, respectively, corresponding to 1% FDR. In liver, we identified a total of 3,599 local eQTLs and 3,912 distant eQTLs at p-values of 9.0×10^−4^ and 1.6×10^−6^, respectively, corresponding to 1% FDR. Plots in which the location of the significantly associated SNP is graphed against the location of the regulated gene are presented in **S11 Fig (liver)** and **S12 Fig (aorta)**.

To prioritize candidate genes in the atherosclerosis loci discussed above, we examined each of the annotated genes for local eQTL. Three of the 4 loci were associated with the expression levels of local genes (2Mb on either side of the locus) in aorta and liver (**S7 Table**). For example, in the Chr 9 locus, examination of hepatic eQTL identified 4 candidates; *Nnmt* (p = 1.27×10^−8^), *2310030G06Rik* (p = 5.42×10^−7^), *Alg9* (p = 1.05×10^−6^), and *1110032A03Rik* (p = 2.27×10^−6^), whose eQTL are regulated by a SNP in high LD (r^2^>0.5) with the associated SNP for atherosclerosis. The less stringent cutoff for r^2^ was chosen to ensure that that no strong eQTL contributing to the atherosclerosis phenotype would be missed, based on the rationale that the causal gene might reside immediately outside the LD block but be regulated by an enhancer within the block. The expression of all four of these genes had significant correlations with lesion size (r = 0.25–0.37, p = 1.2×10^−2^–1.9×10^−4^). Three of the genes also exhibited significant eQTL in aorta; *Nnmt* (p = 1.01×10^−11^), *Bco2* (p = 1.61×10^−4^), *1110032A03Rik* (p = 2.18×10^−4^), and their expression levels were correlated with atherosclerosis (r = 0.26–0.39, p = 2.6×10^−2^–6.4×10^−4^) (**Panel B** in **S11 Fig**). In both tissues, *Nnmt* had the most significant local eQTL (**Panel B** in **S11 Fig** and **Panel B** in **S12 Fig**). In the Chr 5 locus, there are 4 hepatic genes with eQTL that are in strong LD with the atherosclerosis associated SNP at the locus: *Nub1* (p = 1.65×10^−16^ *)*, *Nos3*, (p = 3.08×10^−6^), *Cdk5* (p = 2.48×10^−5^), and *Abcb8* (p = 7.46×10^−4^) (**S5 Fig**). Of these genes, only *Nub1* replicates in the aorta (p = 1.37×10^−15^). While the distal Chr 2 locus was associated with the expression level of two genes in liver, *Dtwd1* (p = 1.43×10^−13^) and *Bub1* (p = 4.62×10^−4^), their abundances were not correlated with lesion size (r = 0.02–0.08, p = 0.43–0.85). None of the loci appear to exhibit interactions with any risk factors examined in this study, including plasma lipids, metabolites, or the cytokines.

### Heritability and contributions of risk factors to atherosclerosis

“Heritability” is the fraction of trait variance that is due to genetic factors [57]. We estimated the heritability of the traits in our study using two different approaches, one that determines heritability due to additive genetic variance, termed “narrow sense heritability” [58] and the second that estimates total heritability, termed “broad sense heritability”. Broad sense heritability was calculated using an R package [59]. The estimates of narrow sense heritability were based on sharing of genomic regions identical by descent. We have recently performed high density genotyping of all the HMDP strains [56] and we used these data to determine genome sharing. Broad sense heritability was estimated based on the reproducibility of trait measurements in different individuals of the strain, a measure of the trait variance due to environmental factors. This estimate includes non-additive factors such as dominance and gene-by-gene interactions.

The resulting heritabilities are shown in **Table 2**. Particularly noteworthy is the fact that, in the case of atherosclerotic lesion area, the broad sense heritability is much larger than the narrow sense heritability (0.63 vs. 0.31), suggesting that non-additive factors such as gene-by-gene interactions are important. Simple inspection of the F1 atherosclerosis data supports this conclusion. Since all the mice are F1 heterozygotes with C57BL/6J (which have lesions of about 150,000 μm^2^ in females and 19,000 μm^2^ in males) as one parent, an additive model would be inconsistent with lesion areas less than about 75,000 μm^2^ for females and 9,000 μm^2^ for males. Yet, some strains have lesions areas less than a few thousand μm^2^. Similar evidence of non-additive inheritance was also observed for plasma lipids and a number of other traits studied. (For examples, see **Panel D** in **S1 Fig, Panel C** in **S8 Fig** and **Panel C** in **S10 Fig**) For certain traits, the narrow sense heritability exceed the broad sense heritability (**Table 2**). This undoubtedly reflects errors in the estimates. In particular, certain traits were examined in only a subset of the mice, compromising estimates of broad sense heritability that are based on reproducibility of measurements within a strain. Overall, these heritability estimates are somewhat lower than observed in some previous HMDP studies [15]. This may be due in part to the fact that the mice were F1 heterozygotes with C57BL/6J being one of the parental strains, thus reducing genetic diversity. Also, the weak correlation of HDL-C with atherosclerosis may reflect, in part, the low heritability of the HDL-C trait.

**Table 2:** Heritability of atherosclerosis and risk factor traits in Ath-HMDP.

Metabolite/Phenotype	Narrow-sense Heritability	Broad-sense Heritability
Insulin	0.47	0.30
Glucose	0.38	0.29
Lesion area	0.31	0.63
Arginine	0.28	0.39
Ornithine	0.25	0.39
Triglycerides	0.25	0.33
Choline	0.22	0.37
Total cholesterol	0.21	0.44
VLDL/LDL	0.20	0.42
Citrulline	0.17	0.30
Trimethylamine	0.16	0.35
TMAO	0.15	0.30
HDL	0.10	0.20

### Application of data to prioritize candidate genes at human GWAS loci

**Fig 11:**
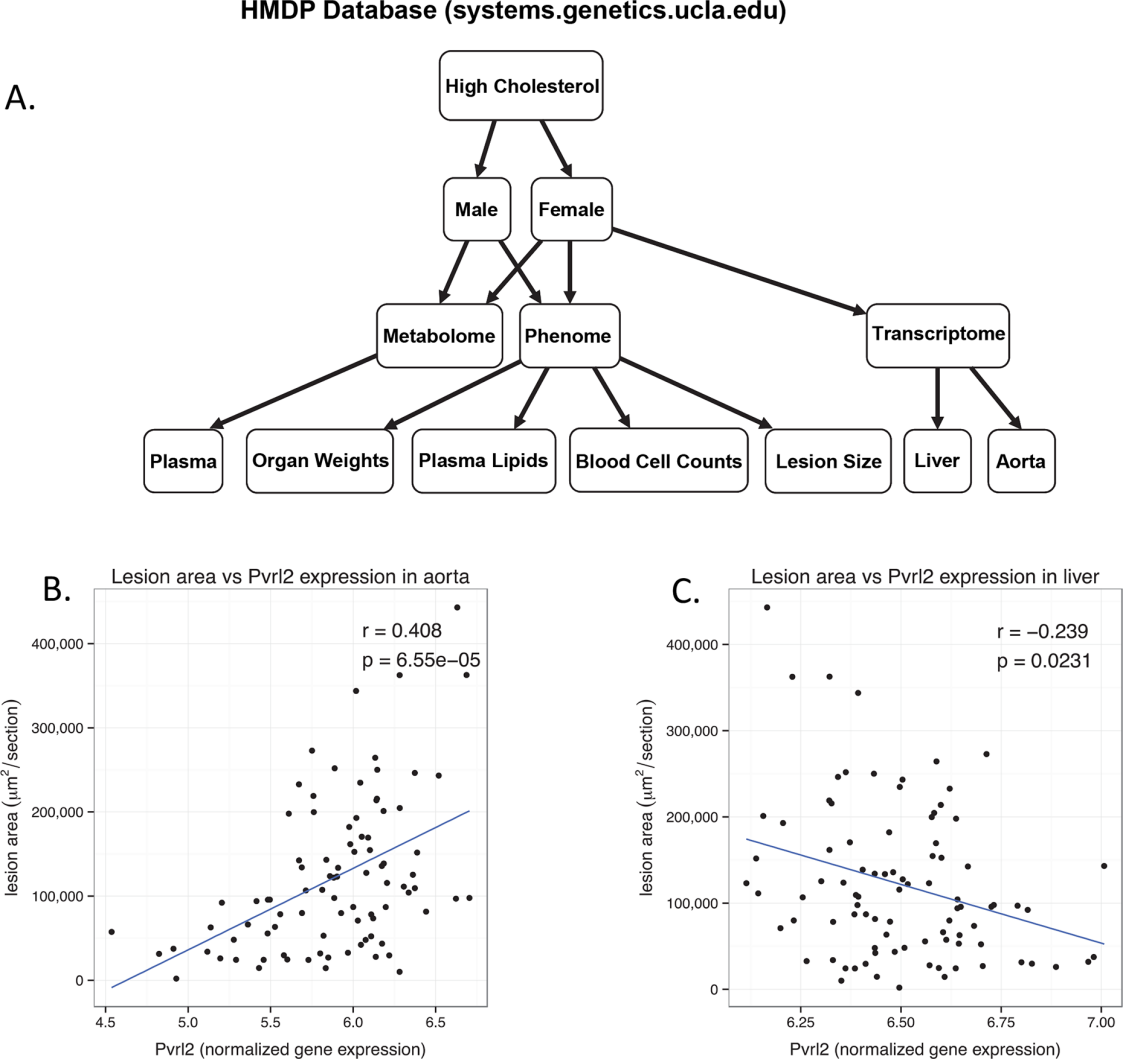
Overview of the systems genetic resource. The database comprises mouse genomic, transcriptomic, metabolomic, proteomic, and clinical trait data from the HMDP as well as selected traditional mouse crosses and several human studies. A hierarchical model of the data and its relationships (**A**). Novel Candidate genes identified in human studies, such as *Pvrl2*, can be interrogated using a variety of data including gene expression in the Aorta (**B**) and liver (**C**) as well as metabolomics and phenotypic traits.

Human GWAS studies employing tens of thousands of individuals are highly powered to identify loci for complex traits, including CAD and its risk factors. To date, about 150 genome-wide significant and suggestive loci have been identified for CAD in approximately 200,000 individuals [60]. Most often, these loci will contain multiple genes in linkage disequilibrium, and thus a first step in further analysis is to identify which of the genes is causally linked to the trait. The data generated in this report can be used for this purpose provided that the human candidate has a functional genetic variation in the mouse (**S8 Table**). For example, the GUCY1B3 gene locus has been associated with atherosclerosis and its role has been validated by studies of rare variants in human families [60–62]. In the *Ath*-HMDP, *Gucy1b3* has a strong *cis*-eQTL in aorta (p = 9.7x10^-6^) as well as other tissues and its expression in aorta is negatively associated with atherosclerosis (r = 0.23, p = 0.04), consistent with human studies. In some cases, our data suggest that a novel gene at a GWAS locus might be responsible. For example, human SNP rs2075650 in Chr 19 has been associated with increased CAD risk and is near the *APOE/APOC1* genes whose roles in atherosclerosis are well established. However, our data show a very significant correlation between the aortic expression of another gene located at the same locus, *Pvrl2*, and lesion size (r = 0.55, p = 3.0×10^−7^) **(Fig 11B)**. There is also a weaker but significant correlation in the liver in the opposite direction (r = -0.27, p = 0.007) (**Fig 11C, S6 Table**). This gene encodes a protein that is part of the adherens junctions [63], and PVRL2 mRNA and protein were shown to be elevated in the vessel wall of diseased human carotid arteries [64] and lesioned mouse aortas [65]. In addition to genes identified by GWAS approaches, the data from the Ath-HMDP should be useful to identify rare variants affecting atherosclerosis. For example, recent exome sequencing analysis of a highly affected family identified mutations in the gene CCT7 that impairs guanylyl cyclase signaling and increased risk of myocardial infarction [62] and its role has been validated in mouse knockout studies [60, 61]. Our data show very significant correlations between aortic expression of *Cct7* and atherosclerosis (r = -0.39, p = 6.1x10^-4^) (**S6 Table)** while hepatic expression was not correlated with lesion size (r = 0.003, p = 0.97).

### Stepwise linear regression analysis of plasma metabolites: Diagnostic markers of atherosclerosis

Our systems genetics approach, in which a variety of plasma metabolites were measured across a population varying for atherosclerosis while holding environmental factors relatively constant, enables the identification of diagnostic markers based on genetic variation. Using a series of plasma metabolites that exhibited at least suggestive correlation with atherosclerosis, we carried out stepwise linear regression [66] to estimate the percent variation in lesion area attributable to each (**Table 3**). These metabolites explain a very significant fraction of disease variance, 31.8% in females and 38.9% in males. One striking difference between the sexes is the lack of impact of TMAO in males. This is likely explained by the fact that male mice show greatly reduced TMAO levels due to repression by testosterone of FMO3, the enzyme that metabolizes TMA to TMAO [67]. The explanation for the sex difference in butyryl-carnitine is unclear as its levels are similar in both sexes. Arginine is strongly associated with lesion area in both sexes presumably reflecting its relationship to NO biosynthesis [68]. It is also of interest that, in this study, HDL-cholesterol levels do not appear to be a significant factor, consistent with Mendelian randomization studies which suggest that HDL-cholesterol levels are not causal for the disease [69].

**Table 3:** Stepwise linear regression for plasma metabolites associated with atherosclerotic lesion area.

Sex	Predictor	Variance explained (%)	
***Female***	Butyryl-carnitine	9.1	
	TMAO	7.8	
	VLDL+LDL	5.7	
	HOMA-IR	4.1	
	Arginine	3.1	
	Insulin	2.0	
		*Female total %*	*31*.*8*
***Male***	VLDL+LDL	20.2	
	Arginine	8.1	
	HOMA-IR	4.4	
	Citrulline	2.6	
	Glucose	2.0	
	Insulin	1.6	
		*Male total %*	*38*.*9*

## Discussion

Over the last 35 years several groups, including ours, have investigated common genetic variations affecting atherosclerosis in mice. In the current study we have examined susceptibility to atherosclerosis and a number of related traits on a hyperlipidemic background in >1,800 mice across 100 strains. The results provide a broad view of the physiological and molecular interactions underlying atherosclerosis in this model organism. A number of conclusions have emerged, as discussed below, and the data reported here should serve as a resource for future gene discovery and mechanistic studies. Toward this end, all of our data can be accessed at our website (systemsgenetics.ucla.edu) or from the authors.

Using association rather than linkage analysis, we have mapped loci for a number of clinically relevant traits with excellent resolution. Of particular interest are traits such as TMAO levels which have proven difficult to address using human GWAS studies, presumably due to the major impact of environmental factors [70]. Although the underlying genes may differ between mice and humans (for example, we did not observe overlap between the four atherosclerosis loci in our mouse population and human GWAS loci for CAD), there appears to be conservation of the underlying pathways. For example, we find a high level of concordance of the major risk factors for atherosclerosis in mice and humans, including the levels of plasma LDL-cholesterol and TMAO. Because our population of mouse strains is relatively small (as compared to human GWAS studies), we are powered to identify only the loci with largest effect sizes (several percent of total trait variance) using GWAS [12]. A complementary approach for understanding pathways contributing to complex traits in mice is “systems genetics” (or “integrative genetics”) [10]. This approach utilizes correlation and mathematical modeling of multi-level phenotypic data to help identify the underlying pathways. We have carried out preliminary systems genetics analyses of these data, including enrichment of annotated biologic pathways in the genes most correlated with clinical traits and co-expression network modeling. Such analyses are difficult to perform directly in human subjects because of the difficulty of accessing tissues such as blood vessels and liver.

Using our systems genetics approach we identify several important characteristics of atherosclerosis. One is its sheer complexity. We carefully phenotyped the Ath-HMDP for a variety of traits and prioritized these based on their correlation to atherosclerosis. These included plasma lipids, metabolites, and cytokines and cellular uptake of modified lipids by thioglycollate-elicited macrophages. Each of these phenotypes are themselves complex traits and we identify significant loci for each of them. Our results are consistent with an infinitesimal model of common diseases, in which genetic variations in hundreds or thousands of genes determine genetic susceptibility to disease.

There were several surprising findings in this study. The first is the importance of non-additive genetic variance in atherosclerosis and some other traits. This was clearly reflected in the F1 data and in the comparison of broad sense as compared to narrow sense heritability. This indicates the existence of important non-additive interactions, such as gene-by-gene and gene-by-environment interactions, which have been difficult to pursue in human studies [71, 72]. Our overall estimates of heritability are similar to estimates in human populations, generally in the range of 0.4 to 0.5 as reviewed previously [73]. We also note the dramatic differences in lesion size between male and female mice. We and others have observed sexually dimorphic results in previous genetic crosses but not to the extent observed in the current study [32, 74–76]. Understanding how genetic and hormonal factors affect lesion development could identify gender specific susceptibility pathways.

Consistent pathways were identified in our gene set enrichment analyses, a primary one being the NO pathway. The endothelial nitric oxide synthase gene (e*NOS*), a strong candidate at the Chr 5 locus, and plasma levels of arginine, the substrate for NO production, are correlated with atherosclerosis and are regulated by a locus on Chr 7 that contains the gene *Nosip1*. Further understanding how NO metabolism is regulated may identify novel targets for atherosclerosis treatment. A number of human epidemiologic studies have revealed associations between arginine and asymmetrical dimethyl arginine, an endogenous NO inhibitor, and cardiovascular events, including myocardial infarction and stroke [77–79].

In addition to arginine, our metabolomics analyses revealed associations between atherosclerosis and several plasma metabolites. The association between TMAO and atherosclerosis adds to the previous evidence that TMAO is involved in atherosclerosis. It is noteworthy that TMAO was a significant factor only in females, which have significantly higher levels of TMAO than males due to repression of FMO3 by testosterone [67]. Briefly, TMAO is derived from dietary choline or carnitine which are metabolized by gut microbiota to trimethylamine. This is, in turn, absorbed into the circulation and oxidized in liver by FMO3 to TMAO [45, 67]. In addition, we found that the levels of choline were inversely associated with atherosclerosis. The negative association with choline could be due to differences between strains in the catabolism of choline to trimethylamine by gut bacteria. Those strains exhibiting increased catabolism would exhibit elevated TMAO production and increased atherosclerosis. By contrast, in humans where differences in plasma choline levels are likely determined primarily by dietary intake, the positive correlation between plasma choline and atherosclerosis is consistent with increased TMAO derived from higher levels of choline substrate [23]. The loci identified for TMAO levels did not contain any obvious candidate genes. It is possible that the loci for TMAO actually influence the composition of gut bacteria responsible for choline or carnitine catabolism. The explanation for the association of butyryl-carnitine with atherosclerosis is unclear. Elevated levels of the metabolite are associated with rare forms of short-chain acyl-CoA dehydrogenase deficiencies [80].

*In-vitro* lipid loading of macrophages using AcLDL has been widely used as a surrogate assay for foam cell formation, a critical event in atherosclerotic lesion formation [81]. The studies performed here were designed to examine this trait in the context of common genetic variation. While we did identify loci associated with lipid loading, they are distinct from loci identified as regulating atherosclerosis in the Ath-HMDP. Nor did the loci correspond to any known genes involved in cholesterol transport, such as scavenger receptor A1 or CD36, which have been shown to affect atherosclerosis in some loss of function studies [82, 83] but not others [84]. Furthermore we did not observe a correlation between lipid loading and atherosclerosis susceptibility, suggesting either that AcLDL is a poor surrogate for the modified, aggregated LDL that is produced in atherosclerotic lesions or that the rate of lipid loading is not limiting for atherosclerosis development. These identified loci may, however, be of basic interest with respect to pathways contributing to cholesterol metabolism and transport. While LDL that has been oxidized *in-vitro* (oxLDL) would be a more suitable ligand for these studies, we used AcLDL for our experiments because there are large variations in uptake between batches of oxLDL as well as variations in the same oxLDL batch over time. A previous study of AcLDL loading of macrophages from two strains differing markedly in atherosclerosis susceptibility (DBA/2J and AKR/J) observed some striking differences in transcriptional responses associated with lysosome and ER stress pathways [85].

Inflammation is a hallmark of atherosclerosis [86] and cytokines and chemokines have been directly linked to lesion development using gain or loss of function studies in mice [87]. Our results revealed significant associations between atherosclerosis and the plasma levels of the cytokine KC (keratinocyte-derived chemokine), a ligand for *Cxcr2* and encoded by the gene *Cxcl1*, located on Chr 5. The homolog of KC in humans is IL-8, a critical cytokine that is elevated in CAD patients [88] and also predictive of future events in apparently healthy subjects [89]. Mechanistic studies in mice have demonstrated that increased expression of KC promotes atherosclerosis by altering monocyte and neutrophil accumulation [90], probably reflecting KC’s (IL-8’s) role in monocyte and neutrophil recruitment [91, 92]**.**

GWAS analysis of KC/IL-8 levels in the Ath-HMDP mice identified a locus on Chr 1 with a large effect within a locus previously identified for a variety of cardiovascular risk factors including atherosclerosis, body weight, and plasma levels of HDL, glucose and triglycerides. The *Apoa2* gene has been identified as the causal variant underlying the lipid variations at this locus [93] but it is not clear that genetic variation in *ApoA2* is directly responsible for all of these phenotypes. We note several positional candidates for this locus including *Slamf7* and *Slamf6* (members of the Signaling Lymphocyte Activation Molecules family that mediate NK cell activation) and *Ifi203* and *Ifi204* (interferon activated genes 203 and 204). Of these, only *Ifi203* has an eQTL (3.2×10^−24^) regulated by a SNP in high linkage disequilibrium (r^2^>0.5) with the associated SNP for KC. It also has expression levels significantly correlated with KC levels (p = 0.003).

We also analyzed blood cell levels in the Ath-HMDP mice and observed that the number of monocytes as percent of leukocytes was significantly associated with atherosclerosis. This is consistent with human population studies showing that increased monocyte numbers are associated with disease [94]. We also identified loci, associated with monocyte levels; it is noteworthy that these differed from those previously identified in mice on chow diets [95].

Finally, our data constitute a resource for elucidating pathways contributing to atherosclerosis. For example, they should be useful in prioritizing candidate genes at human GWAS loci for atherosclerosis and related traits. More than a third of the genes present on the arrays used here exhibited significant genetic variation in expression in aorta and liver. For example we show that the vascular expression of *Pvrl2*, a positional candidate from human GWAS studies, is highly correlated with atherosclerosis. Another example is the strong association of expression levels of Cdkn2b with atherosclerosis (**S8 Table**). This gene resides in the human chromosome 9p21 region that is the most strongly associated with atherosclerosis and our data suggest that it may have a causal role [96]. The resource is also being expanded to include additional expression, metabolome, and microbiome data (in progress).

In conclusion, our study provides a comprehensive systems genetic analysis of traits relevant to atherosclerosis in a population of common inbred strains of mice. The results are generally consistent with human epidemiologic studies, as many of the factors associated with atherosclerosis in human populations were replicated in mice. They also identify a number of novel factors and candidate gens that can now be experimentally examined. These data as well as other HMDP studies, including gene-gene and gene-trait correlations and clinical trait and clinical trait and transcript mapping, can be accessed in a user-friendly web-based interface at http://systems.genetics.ucla.edu/data.

## Materials and Methods

### Online database

Results can be accessed at http://systems.genetics.ucla.edu/data

### Accession numbers

All microarray data from this study are deposited in the NCBI GEO (http://www.ncbi.nlm.nih.gov/geo/) under the accession number GSE66570.

### Animal studies

Mice carrying the human transgene for cholesteryl ester transfer protein (CETP) on a C57BL/6 background were obtained from The Jackson Laboratory (Stock Number:003904). Mice carrying the human ApoE3 Leiden variant were kindly provided by Dr. L. Havekes [97]. For purposes of these experiments, we interbred these mice to create a strain carrying both transgenes and these were bred to females from about 100 common inbred and recombinant inbred strains purchased from The Jackson Laboratory. The locations and copy numbers for the transgenes are unknown. However, while there may be insertion effects on expression of the transgenes or neighboring genes, all F1 animals received their transgenes from the same transgenic mice so that such insertion effects should be uniform across the Ath-HMDP panel. Male and female progeny were genotyped for the presence of both transgenes and, at the age of about 8 weeks, were placed on a “Western Style” synthetic high fat diet (33 kcal % fat from cocoa butter) supplemented with 1% cholesterol (Research Diets D10042101) (**see S9 Table**). After 16 weeks on this diet, animals were euthanized for the collection of tissue. Animals were maintained on a 12hr light-dark cycle, 6AM-6PM with *ad libitum* access to water and chow or experimental diet. Euthanasia of all mice was carried out using deep anesthesia with isoflurane vapor followed by cervical dislocation, a procedure consistent with recommendations of the AVA.

All animal work was conducted according to relevant national and international guidelines and was approved by the UCLA Animal Research Committee, the UCLA IACUC.

### Plasma collection

Plasma was collected from the retro orbital plexus under isoflurane vapor anesthesia immediately before starting the experimental diet at about 8 weeks of age and again at the time of euthanasia at approximately 24 weeks of age. In both cases, the animals were fasted for 4h beginning at 6AM. Blood was collected using heparinized glass capillary tubes into plasma collection tubes with EDTA (Becton Dickerson). Blood was kept on ice until centrifuged and the separated plasmas were frozen at -80°C in aliquots for subsequent analysis.

### Plasma phenotypes

Plasma lipid profiles were measured by colorimetric analysis as previously described [98, 99]. Quantification of plasma cytokines was carried out in a multiplexed immune-capture microbead system (Milliplex Mouse Cytokine / Chemokine Magnetic Bead Panel MCYTOMAG-70K (EMD Millipore, Billerica, MA)) as per manufacturer’s instructions. Cytokines profiled were: G-CSF, GM-CSF, IFNr, IL-1α, IL-1β, IL-2, IL-4, IL-6, IL-7, IL-10, IL-12 (p40), IL-12 (p70), IL-13, IL-15, IP-10, KC, MCP-1, MIP-1α, MIP-1β, M-CSF, MIP-2, MIG, RANTES and TNFα. Plasma insulin was measured using the mouse insulin ELISA kit (80-INSMS-E01) from Alpco (Salem, New Hampshire) as per manufacturer’s instructions. Blood for hematology analysis was collected from mice from the retro-orbital plexus under isoflurane anesthesia. Complete blood cell profiling was carried out using the Heska (Loveland, CO) HemaTrue(TM) Veterinary Hematology Analyzer. Blood was collected in 20 μl EDTA-coated glass capillaries and processed using standard procedures as per instructions from Heska.

### Atherosclerotic lesions

Lesion area in the proximal aorta was carried out as previously described [93, 100]. Briefly, the aorta was flushed with PBS and embedded in OCT. Frozen sections (10 μm) were stained with Oil Red O and lesion area quantified in every 3^rd^ section through the proximal aorta. Lesion size was not normally distributed and was transformed using the Yeo-Johnson transformation [101].

For immunohistochemical staining, primary antibody for α-smooth muscle actin (α-SMA) (Rabbit anti alpha smooth muscle actin abcam # ab32575) or CD68 (for macrophages; Rat anti–mouse CD68, Bio-Rad # MCA 1957) was applied on tissue sections from frozen, OCT-embedded proximal aortas. Frozen tissue sections were fixed in acetone (15min at 4°C), washed, blocked with 5% normal goat serum in 3% BSA (3h at room temperature), washed with PBS and incubated with the primary antibody for 1 h at room temperature and then overnight at 4°C. After washing with PBS, secondary antibody (α-SMA: Goat anti rabbit (Vector cat # BA-1000) or CD68: Goat anti rat (Vector cat # BA-9401)) was added for 1h at room temperature. With intervening washes, slides were then treated with ABC solution (Vector Laboratories Cat# AK-5000, 1h at room temperature), Vector red substrate (Vector Cat# SK-5100, 10-60min), Hematoxylin (Thermoscientific #7211, 2 min), Bluing reagent (Thermoscientific #7301, 1–2 minutes) and 0.01% fast green (Sigma #F7258, 30–60 seconds) and then dried and mounted with glycerol gelatin solution from Sigma (Cat# GG1). Slides stained for CD-68 macrophages, or for α-smooth muscle actin were analyzed for percent of lesion area with positive immuno-reactivity based on visual scoring of a superimposed grid.

### Plasma metabolites

We measured the following metabolites using MS/MS TOF as described [102]: acetyl-carnitine, arginine, asymmetric dimethylarginine (ADMA), betaine, butyrobetaine, butyryl-carnitine, carnitine, choline, citrulline, creatinine, crotonobetaine, gamma-butyrobetaine, hexanoyl-carnitine, isoleucine, leucine, Lysine, methyllysine, monomethylarginine (MMA), octenoyl-carnitine, ornithine, pentanoyl-carnitine, phenylalanine, propionyl-carnitine, symmetric dimethylarginine (SDMA), trans-crotonobetaine, trimethylamine (TMA), trimethylamine N-oxide (TMAO), tyrosine and valine.

### LC/MS/MS methodology

Stable isotope dilution LC/MS/MS was used for quantification of plasma analytes. Four volumes of methanol containing isotope-labeled internal standards were added to 1 volume of plasma to precipitate protein. The supernatant after centrifugation was analyzed by injection onto a silica column interfaced with an API 4000 Q-TRAP mass spectrometer (AB SCIEX, Framingham, MA) [102]. A discontinuous gradient was generated to resolve the analytes by mixing solvent A (0.1% propanoic acid in water) with solvent B (0.1% acetic acid in methanol) [102]. Analytes and the isotope labeled internal standards were monitored in positive MRM MS mode using characteristic precursor–product ion transitions. The parameters for the ion monitoring were optimized for each analyte. Various concentrations of analytes were spiked into the control plasma sample to prepare the calibration curves for quantification of analytes.

### Body composition

Body composition was measured a day or two before euthanasia by NMR using the Brüker minispec (Brüker Biospin Corp, Billerica, MA) and software from Echo MRI (Houston, TX) [103].

### Gene expression in the aorta

Whole aorta from the arch to the mid-abdomen was cleaned of peri-adventitial adipose and snap-frozen at the time of euthanasia, and total RNA isolated using the Qiagen (Valencia, CA) RNeasy kit, as described [104]. Genome wide expression profiles were determined by hybridization to Affymetrix HT-MG_430 PM microarrays on a subset of female mice from 104 strains (N = 1 to 10 aorta per strain).

### Gene expression in the liver

The liver was carefully dissected and a 50-μg aliquot from the left lobe was immediately frozen at the time of euthanasia and total RNA isolated using the Qiagen (Valencia, CA) RNeasy kit (cat# 74104), as described [104]. Genome wide expression profiles were determined by hybridization to Affymetrix HT-MG_430 PM microarrays on a subset of female mice from 96 strains (N = 1 to 3 liver samples per strain). To assess reliability of results from these microarrays, we compared expression in livers of Ath-HMDP F1 mice carrying a recipient genome from C57Bl/6J, A/J, DBA/2J or BALB/cJ with RNA-seq data for the same strains (n = 3 mice per strain). The correlation between these two approaches was quite strong, (approximately r = 0.72, p< 1.x10^-16^ for each strain) similar to a recently published comparison of microarray and RNA-seq data [105].

### Macrophage LDL uptake

Primary macrophages were harvested from four mice per strain, by intraperitoneal lavage four days following intraperitoneal injection of 1.5ml 4%Thioglycollate (BD, Sparks, MD). All mice were injected with the same batch of thioglycollate. Cells from each strain were pooled, and plated in replicate wells (n≥ 4) of 96-well black plates (Fisher, Pittsburgh, PA) at a cell density of 3×10^5^ cells per well in DMEM with 20% FBS at 37°C and 5% CO_2_. After overnight culture, cell media was replaced with 1% FBS DMEM media for controls or with media plus 10μg/mL DiI-acetylated LDL (Biomedical Technologies, Ward Hill, MA), or media plus 10μg/mL DiI-acetylated LDL and 200μg/mL unlabeled acetylated LDL (Biomedical Technologies). Four hours later, these media were removed and wells were washed 3 times with PBS and measured for DiI fluorescence (Excitation at 530 nm; Emission at 590 nm)**.**

### Weighted Gene Co-expression Network Analysis

Network analysis was performed using the WGCNA R package [52]. An extensive overview of WGCNA, including numerous tutorials, can be found at http://www.genetics.ucla.edu/labs/horvath/Co-expressionNetwork/ and this method has been extensively used to create co-expression networks [52, 106–110]. To generate a co-expression network for all probes, an adjacency matrix is created by first calculating the pairwise gene-gene correlations and then raising the Pearson correlation to the 10^th^ and 6^th^ power for aorta and liver, respectively. The power was selected using the scale-free topology criterion, which is determined by the function “pickSoftThreshold” in the WGCNA package [52, 111]. Network connectivity (k.total) of the genes was calculated as the sum of the connection strengths with all other network genes. A TOM-based dissimilarity measure was used for hierarchical clustering of the genes. Gene modules corresponded to the branches of the resulting dendogram and were defined using the “Dynamic Hybrid” branch cutting algorithm [112]. The parameters for module generation were as follows: “cut height” parameter was set to 0.99 and the “minimum module size” parameter was set to 30. Gene significance (GS) for each gene was determined and is defined as the correlation between lesion size and expression of probes. Module significance (MS) was calculated as the mean GS for all module genes. Modules that were most significantly correlated with lesion size in aorta and liver were visualized using Cytoscape [113].

### Gene ontology

We performed a Gene Ontology (GO) enrichment analysis for network modules using the Database for Annotation, Visualization and Integrated Discovery (DAVID) using the functional annotation clustering option [114]. Functional annotation clustering combines single categories with a significant overlap in gene content and then assigns an enrichment score (ES; defined as the–log10 of the geometric mean of the unadjusted p-values for each single term in the cluster) to each cluster.

### Association mapping

The Mouse Diversity Genotyping Array [115] was used to genotype ~150 classical and recombinant inbred mouse strains. After eliminating SNPs flagged as having poor quality, approximately 450,000 SNPs formed our starting set of genotypes. These SNPs were then filtered using the following criteria: the minor allele frequency could not be below 10% and the missing genotype frequency could not exceed 10%. Since a different subset of our 150 strains was used in each of our studies (which was dependent on strain availability at the time) this filtering was performed separately for each study.

### Association analysis

Associations were performed using FaST-LMM [55], a linear mixed model method that is able to account for population structure. To improve power, when testing all the SNPs on chromosome N for association, the kinship matrix was constructed using the SNPs from all other chromosomes besides N. This procedure allows us to include the SNP being tested for association in the regression equation only once. Also, any bias from measuring different numbers of mice for a given trait should be accounted for by the kinship matrix of the FaST-LMM algorithm.

### Correlation analysis

The biweight midcorrelation statistic is analogous to the Pearson correlation coefficient, but has the advantage of being robust to outliers. We used the bicor function implemented in the WGCNA R package [116] to calculate transcript-transcript correlations, transcript-trait correlations, and trait-trait correlations. These analyses were performed at the strain level to account for the variable number of mice collected for each trait.

### Local and distant eQTL definition

eQTL were defined as *local* or *cis* if the peak association was within a 4Mb interval, flanking 2Mb on either side of the genomic start site of the gene. eQTL were defined as *distant* or *trans* by selecting the peak association per chromosome per gene, excluding loci that mapped in *cis*.

### Genome-wide alpha for cis-eQTL

We calculate false discovery rates using the *qvalue* package in R. For each gene, we selected all association *p*-values in the 4 Mb interval, and calculated *q*-values using all the *p*-values for all genes. We estimated the FDR separately for each treatment and selected FDR<5%. We estimated the FDR separately for each tissue and selected FDR<5% as follows: aorta eQTL, the median 1% FDR cutoff was 8.4×10^−4^, and 5% FDR cutoff was 6.4×10^−3^. The corresponding results for the liver data were 1% FDR = 9.4×10^−4^ and 5% FDR = 6.7×10^−3^.

### Genome-wide alpha for trans-eQTL

Due to the computational complexity associated with evaluating *q*-values for over 2 billion *p*-values, we computed the FDRs by taking the median FDR for 100 samples, each containing 100 million randomly selected *p*-values from the original calculated association *p*-values [117]. We estimated the FDR separately for each tissue and selected FDR as follows: aorta eQTL, the median 1% FDR cutoff was p ≤ 1.5×10^−6^, and 5% FDR cutoff was 1.3×10^−5^. The corresponding results for the liver data were 1% FDR = 1.6×10^−6^ and p ≤ 5% FDR = 1.30×10^−5^. We set 1.3×10^−5^ as our threshold of significance for all molecular and clinical phenotypes.

### Stepwise linear regression of plasma metabolite levels associated with atherosclerotic lesion area

We used forward stepwise regression [66] to identify those metabolites that appeared to be the most effective predictors of lesion area. To avoid potential issues with over-fitting, we only used as candidate predictors those traits which had at least suggestive correlation with lesion area. These included plasma levels of choline, arginine, butyryl-carnitine, citrulline, TMAO, ornithine, KC, VLDL+LDL, HDL, TG, insulin, glucose, and the calculated parameter HOMA-IR.

## Supporting Information

S1 Fig Plasma lipoproteins in the Ath-HMDP. In each panel, strains are arranged in rank order by strain-average lipoprotein levels ± SEM after 16 weeks on high-fat diet. A) VLDL + LDL cholesterol (mg/dl) in females. B) VLDL + LDL cholesterol (mg/dl) in males. C) HDL cholesterol (mg/dl) in females. D) HDL cholesterol (mg/dl) in males. E) Total cholesterol (mg/dl) in females. F) Total cholesterol (mg/dl) in males. G) Triglycerides (mg/dl) in females. H) Triglycerides (mg/dl) in males.
(PDF)

S2 Fig Plasma lipid correlations between diets. In each panel, strain average plasma lipoprotein levels (mg/dl) for females are plotted for mice on a chow diet at 8 weeks of age (horizontal axis) or after an additional 16 weeks on high-fat diet. A) VLDL + LDL cholesterol, B) Total Cholesterol, C) HDL Cholesterol, D Triglycerides.
(PDF)

S3 Fig Relationships of metabolic risk factors to atherosclerosis in female Ath-HMDP mice. Correlation of atherosclerosis with levels of (**A**) plasma LDL/VLDL-cholesterol (mg/dl), (**B**) plasma HDL-cholesterol (mg/dl), (**C**) plasma triglycerides (mg/dl), (**D**) plasma insulin (pg/ml), (E) plasma glucose (mg/dl) and (F) HOMA-IR. Individual points indicate strain-averages for atherosclerotic lesion area (μm^2^/section) and clinical traits.
(PDF)

S4 Fig Plasma glucose (mg/dl) and insulin (pg/ml) in the Ath-HMDP. In each panel, strains are arranged in rank order by strain-average glucose or insulin levels ± SEM after 16 weeks on high-fat diet. A) Glucose (mg/dl) in females. B) Glucose (mg/dl) in males. C) Insulin (pg/ml) in females. D) Insulin (pg/ml) in males.
(PDF)

S5 Fig High resolution regional plots of atherosclerosis loci in females. LocusZoom plots [89] of atherosclerosis QTLs on (**A**) Chromosome 2 at 93.3 Mb, (**B**) chromosome 2 at 126.6 Mb and (**C**) Chromosome 5 at 24.6 Mb. Significance for association of SNPs with the atherosclerosis phenotype is indicated by–log p-value. Purple diamond indicates position of peak SNP. Other SNP positions are marked as circles with color indicating R^2^ as shown in color scale. Positions of nearby genes are mapped below.
(PDF)

S6 Fig Conditional analysis of atherosclerosis QTLs. To determine if the two QTL peaks on chromosome 2 are independent, association analysis was repeated but conditioning on the peak SNP in each peak. (**A**) Top plot shows dual peaks in absence of conditioning. Middle plot shows association conditioned on peak SNP (rs27381267) at 93.3 Mb while bottom plot shows association conditioned on peak SNP (rs32754652) at 126.6 Mb. In each case, conditioning suppresses association for the local peak but not at the second peak, suggesting that the two association peaks are independent. (**B**) For QTL on chromosome 5, top plot shows broad peaks in absence of conditioning. Bottom plot shows association conditioned on peak SNP (rs32008039) at 24.6 Mb. In this case, conditioning suppresses association across the full region consistent with a single associated locus. Similarly, for QTL on chromosome 9, top plot shows broad peaks in absence of conditioning for both males (**C**) and females (**D**). Bottom plot shows association conditioned on peak SNP (rs33738357) at 49.6 Mb. Again, conditioning suppresses association across the full region consistent with a single associated locus.
(PDF)

S7 Fig Atherosclerosis maping in the Ath-HMDP using only strains with 3 or more mice. Genome wide association plot for atherosclerotic lesion-area restricted to data from strains for which 3 or more females were available. In addition to the previously observed three peaks on chromosomes 2, 5 and 9, there is an additional locus on chromosome 10 that reaches significance.
(PDF)

S8 Fig Plasma cytokine levels in the Ath-HMDP. In each panel, strains are arranged in rank order by strain-average metabolite levels (pg/ml) in females +/- SEM after 16 weeks on Western Diet. (**A**) GM-CSF, (**B**) IL-10, (**C**) MCP-1 (**D**) MIG, (**E**) MIP-1**α** and (**F**) MIP-1**β**.
(PDF)

S9 Fig Reproducibility of DiI-AcLDL uptake in thioglycolate-stimulated macrophages isolated from HMDP mice (relative fluorescence). **(A**) Measurements on different dates for strains AXB13, BXD33, BXD40 and SM. Open circles indicate replicate mice measured on the same day. The closed circles for BXD40 indicate results for replicate mice measured on different days. (**B**) Relative fluorescence after varying concentrations of DiI–Ac-LDL after 4h or DiI-AcLDL plus 200 ug/ml unlabeled-AcLDL (**C**) Relative fluorescence after varying concentrations of DiI–Ac-LDL after 24h or DiI-AcLDL plus 200 ug/ml unlabeled-AcLDL.
(PDF)

S10 Fig Plasma metabolite levels in the Ath-HMDP. In each panel, strains are arranged in rank order by strain-average metabolite levels (uM) in females ± SEM after 16 weeks on Western Diet. **(A)** Butyryl-carnitine, **(B)** Choline, **(C)** Trimethylamine N-oxide (TMAO). **(D)** Arginine, **(E)** Citrulline, **(F)** Ornithine.
(PDF)

S11 Fig Hepatic eQTL analysis in the Ath-HMDP. (**A**) Transcript levels in liver of HMDP mice were profiled and significant associations are plotted according to chromosomal position (*x*-axis) versus the location of the structural gene (*y*-axis). The strong diagonal line represents *cis*-eQTL, whereas the remainder are *trans*-eQTL signals. (**B**) Genome-wide association results in the HMDP demonstrating a strong association for *Nnmt* transcript levels in liver on chromosome 9. (**C)** Correlation between *Nnmt* mRNA levels and atherosclerosis.
(PDF)

S12 Fig Aortic eQTL analysis in the Ath-HMDP. (**A**) Transcript levels in aorta of HMDP mice were profiled and significant associations are plotted according to chromosomal position (*x*-axis) versus the location of the structural gene (*y*-axis). The strong diagonal line represents *cis*-eQTL, whereas the remainder are *trans*-eQTL signals. (**B**) Genome-wide association results in the HMDP demonstrating a strong association for *Nnmt* transcript levels in aorta on chromosome 9. (**C**) Genome-wide association results in the HMDP demonstrating a strong association for *Nub1* transcript levels in aorta on chromosome 5.
(PDF)

S1 Table Correlation of clinical traits with atherosclerosis for females and males. (XLSX)

S2 Table Strain average phenotype measurements for clinical and metabolic traits. For each strain and trait, the number of animals (n), trait average (avg), standard deviation (sd) and standard error of the mean (sem) are given. Separate worksheets are provided for clinical and metabolic traits and for male and female animals.
(XLSX)

S3 Table Clinical trait QTLs. (XLSX)

S4 Table Blood cell correlations with atherosclerotic lesion area. (XLSX)

S5 Table Cytokines quantitated. (XLSX)

S6 Table Top 1000 genes correlating with atherosclerosis. Separate worksheets are presented for genes showing positive or negative expression correlation in aorta or liver.
(XLSX)

S7 Table Local eQTLs and their correlation with lesion size in the atherosclerosis loci. (XLSX)

S8 Table Correlation of the expression of human GWAS gene homologs with atherosclerotic lesion size in aorta and liver (XLSX)

S9 Table Diet composition. (XLSX)
