# Correction: Genetic Architecture of Atherosclerosis in Mice: A Systems Genetics Analysis of Common Inbred Strains

**DOI:** 10.1371/journal.pgen.1005913

**Published:** 2016-03-02

**Authors:** Brian J. Bennett, Richard C. Davis, Mete Civelek, Luz Orozco, Judy Wu, Hannah Qi, Calvin Pan, René R. Sevag Packard, Eleazar Eskin, Mujing Yan, Todd Kirchgessner, Zeneng Wang, Xinmin Li, Jill C. Gregory, Stanley L. Hazen, Peter S. Gargalovic, Aldons J. Lusis

There are errors in the legend for Fig 8. The first sentence “CD68+ cells but not smooth muscle actin+ cells differ among strains comprising the HMDP.” is inaccurate and the sentence has been removed. Differences were found, but these were not significant. This is discussed in the main body of the text.

**Fig 8:**
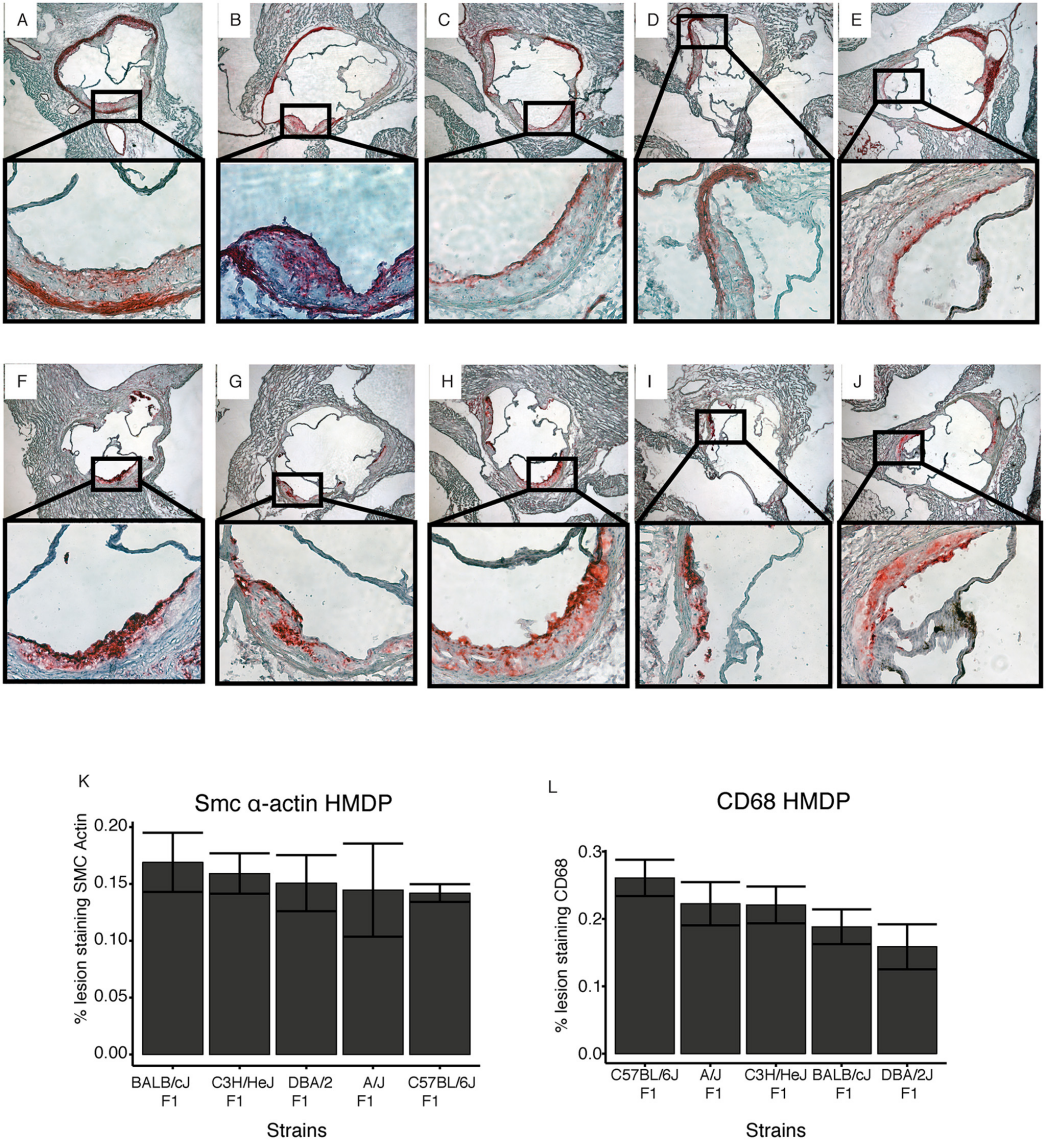
Lesion morphology in 5 progenitor inbred strains. Representative results from lesions immunostained for sm-α actin are shown in Panels **A-E** and for CD68 in panels **F-J**. C57BL/6J (**A,F**), A/J (**B,G**), BALB/cJ (**C, H**), C3H/HeJ (**D, I**), DBA/2J (**E, J**). Panels **K** and **L** show impact of genetic background on lesion morphology in C57BL/6J, A/J, C3H/HeJ, BALB/cJ and DBA/2J, 5 progenitor strains for the HMDP recombinant inbred strains. Immunohistological staining for macrophages (CD68) (**L**) or smooth muscle cells (smooth muscle α-actin) (**K**) expressed as percent of total atherosclerotic lesion area +/- SEM.

The description for panel L incorrectly corresponds to panel K and vice versa. The correct legend is below.
