# Supplementary figures and images for: Genetic Architecture of Atherosclerosis in Mice: A Systems Genetics Analysis of Common Inbred Strains
Source: PLoS Genet. 2015 Dec 22;11(12):e1005711. doi: 10.1371/journal.pgen.1005711 (PMC4687930; doi:10.1371/journal.pgen.1005711)

A. Females

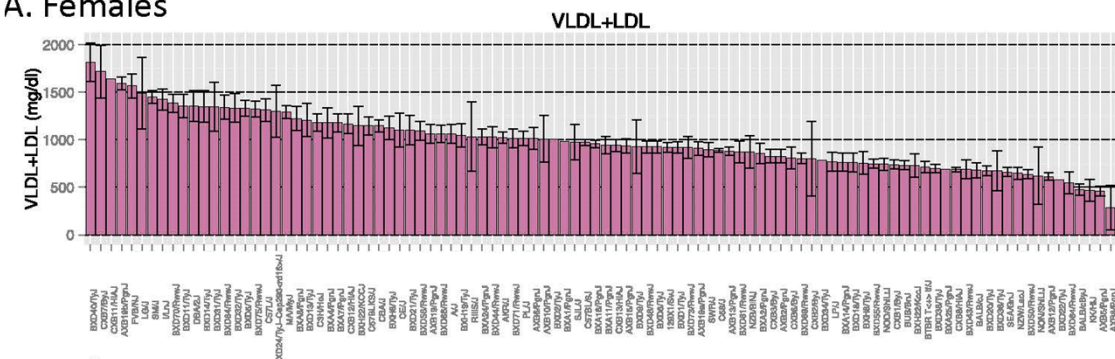

B. Males

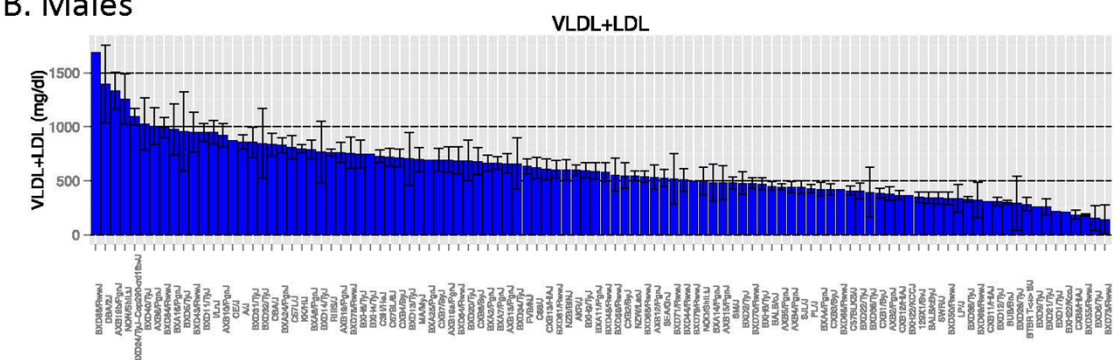

C. Females

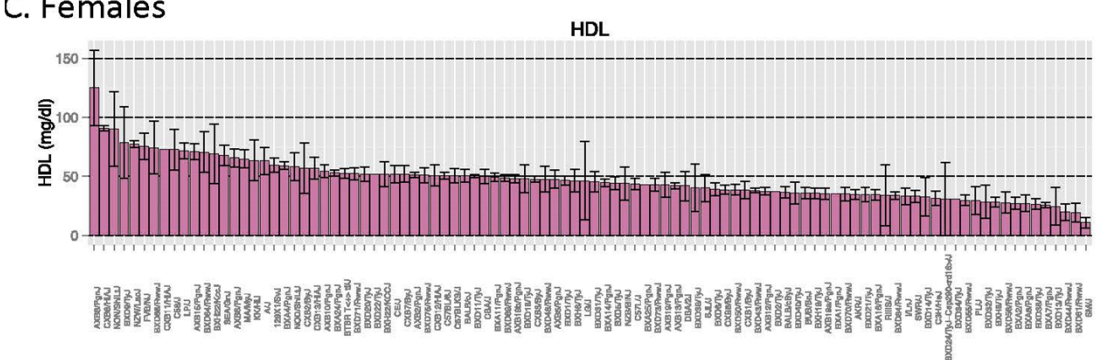

D. Males

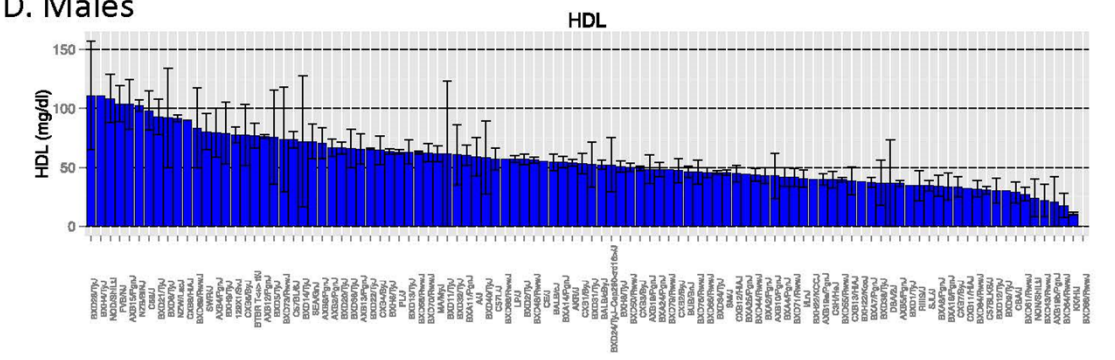

E. Females

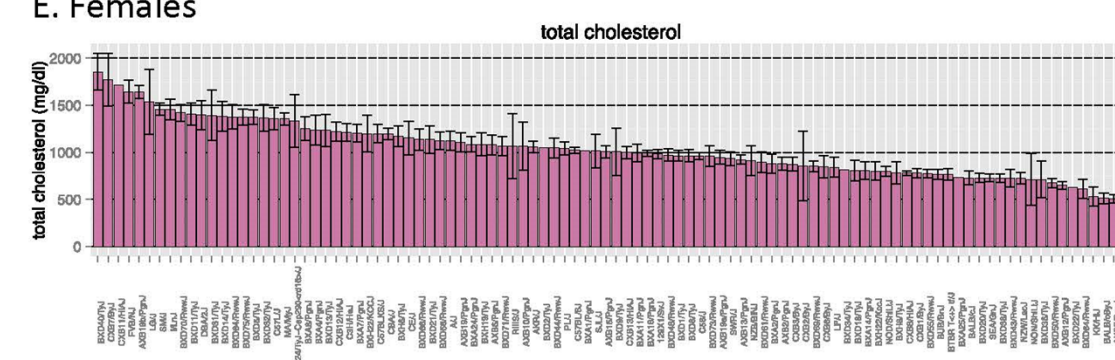

F. Males

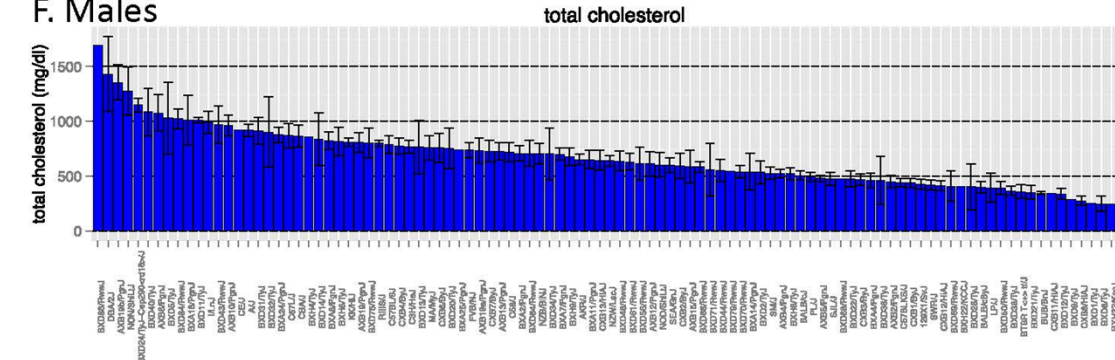

G. Females

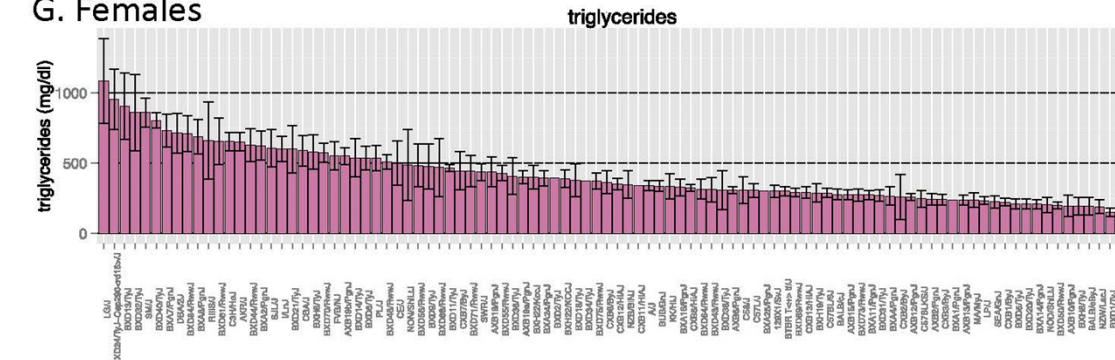

H. Males

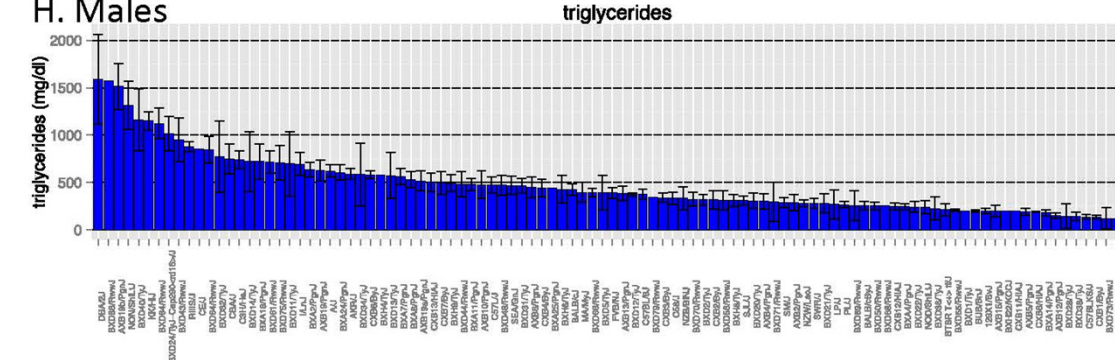

Supplement: S1 Fig — In each panel, strains are arranged in rank order by strain-average lipoprotein levels ± SEM after 16 weeks on high-fat diet. A) VLDL + LDL cholesterol (mg/dl) in females. B) VLDL + LDL cholesterol (mg/dl) in males. C) HDL cholesterol (mg/dl) in females. D) HDL cholesterol (mg/dl) in males. E) Total cholesterol (mg/dl) in females. F) Total cholesterol (mg/dl) in males. G) Triglycerides (mg/dl) in females. H) Triglycerides (mg/dl) in males. (PDF) [file pgen.1005711.s001.pdf]

A.

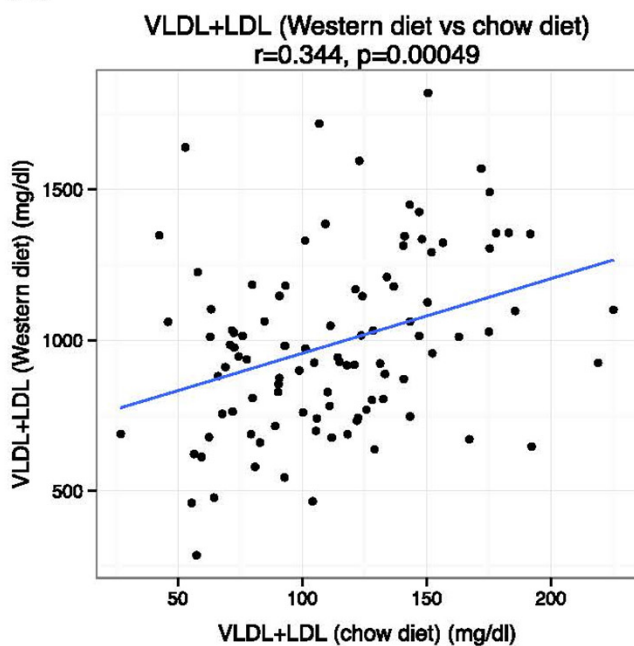

B.

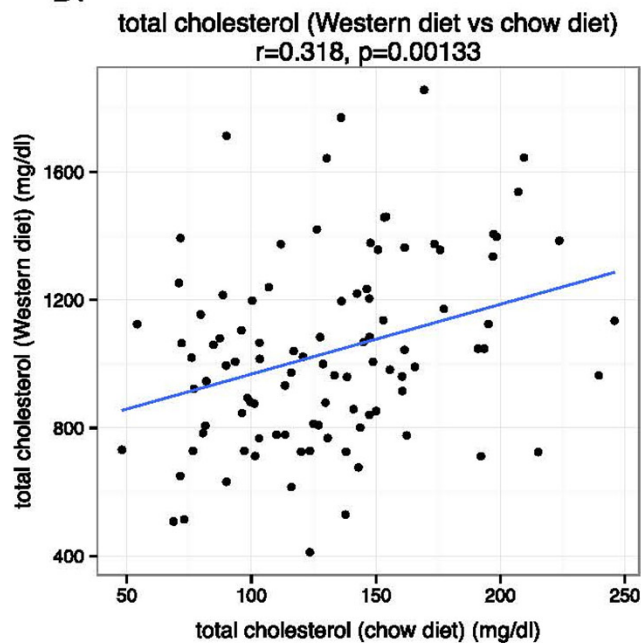

C.

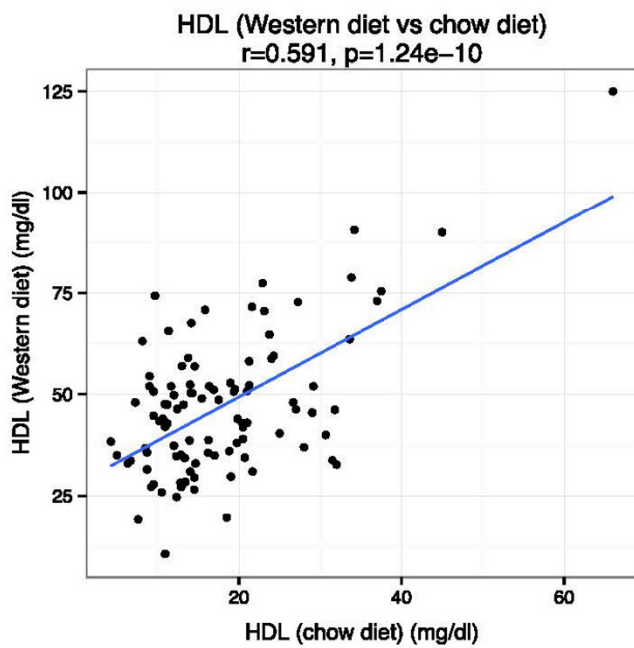

D.

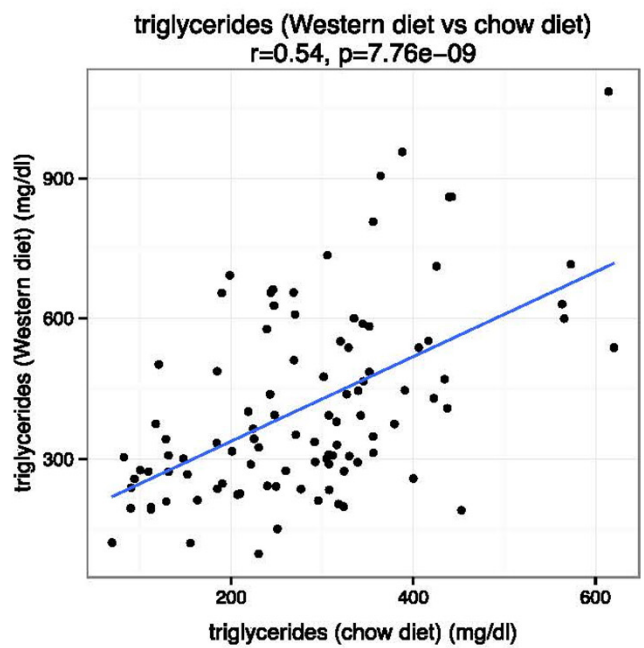

Supplement: S2 Fig — In each panel, strain average plasma lipoprotein levels (mg/dl) for females are plotted for mice on a chow diet at 8 weeks of age (horizontal axis) or after an additional 16 weeks on high-fat diet. A) VLDL + LDL cholesterol, B) Total Cholesterol, C) HDL Cholesterol, D Triglycerides. (PDF) [file pgen.1005711.s002.pdf]

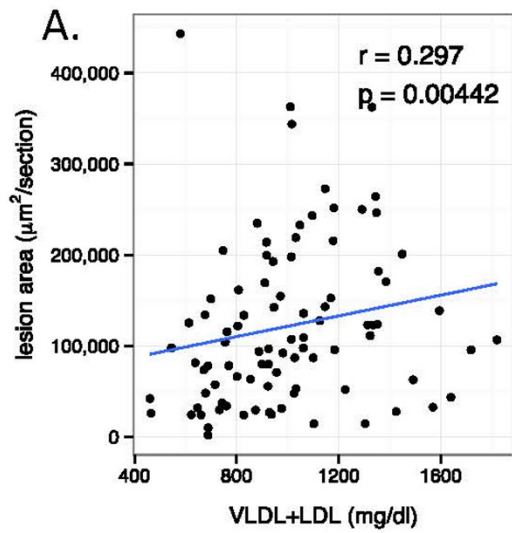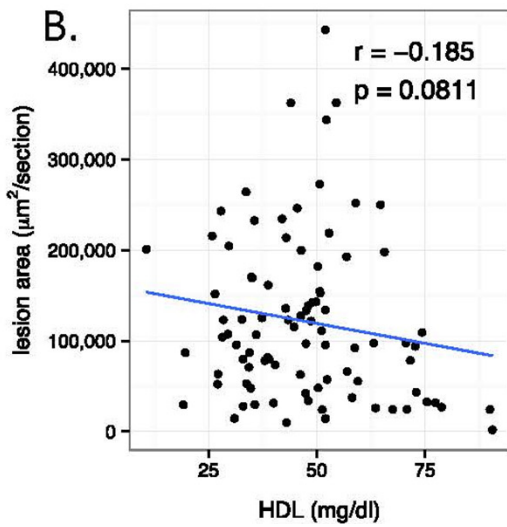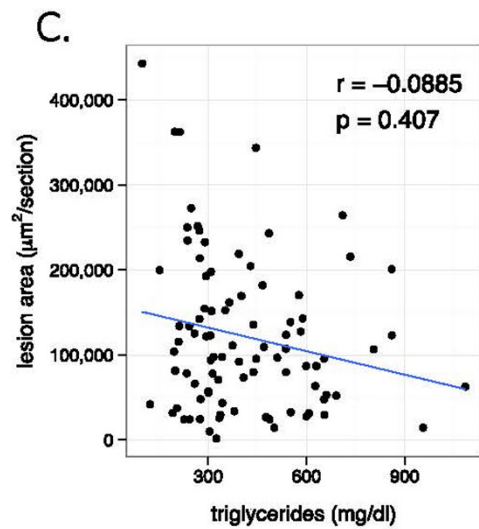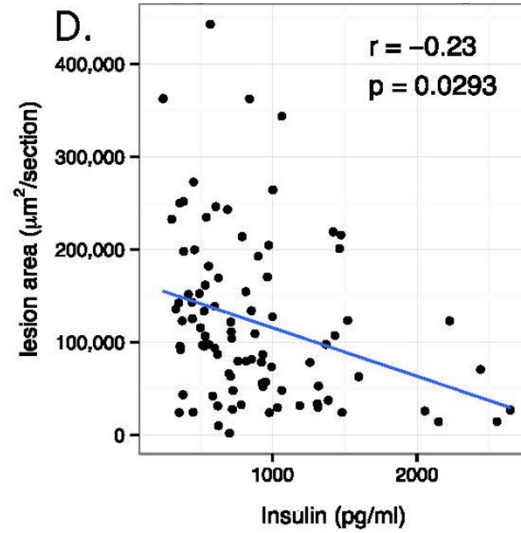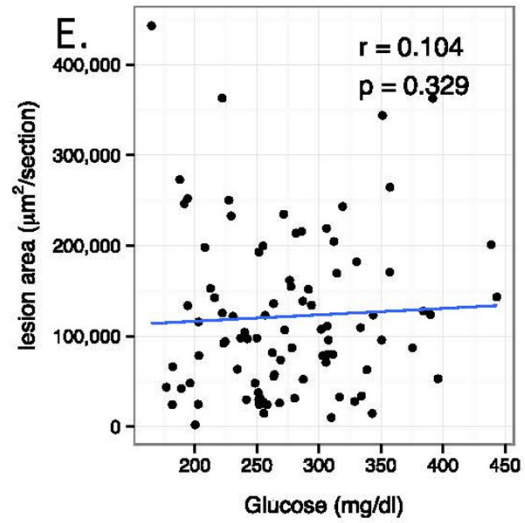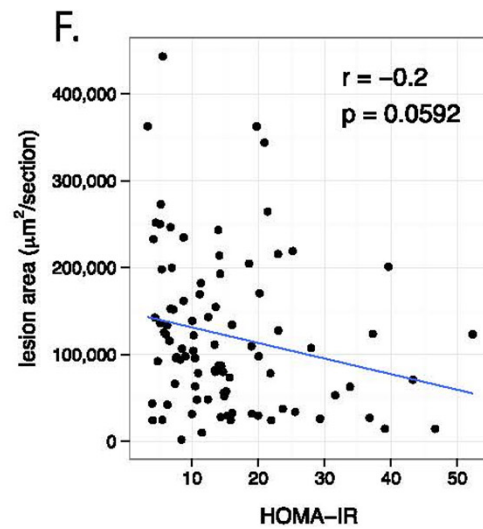

Supplement: S3 Fig — Correlation of atherosclerosis with levels of (A) plasma LDL/VLDL-cholesterol (mg/dl), (B) plasma HDL-cholesterol (mg/dl), (C) plasma triglycerides (mg/dl), (D) plasma insulin (pg/ml), (E) plasma glucose (mg/dl) and (F) HOMA-IR. Individual points indicate strain-averages for atherosclerotic lesion area (μm2/section) and clinical traits. (PDF) [file pgen.1005711.s003.pdf]

### A. Females

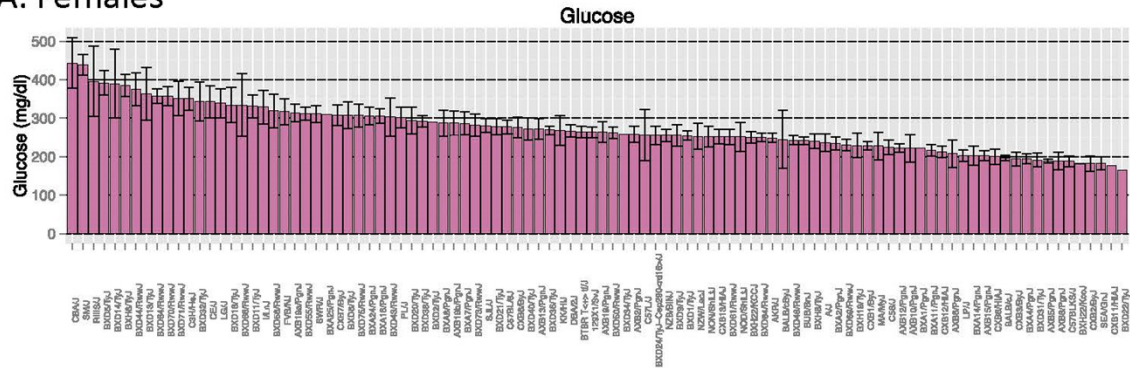

### B. Males

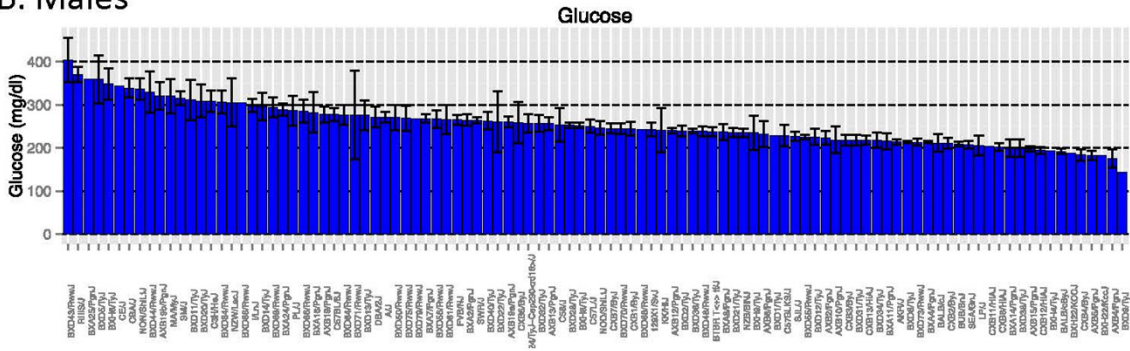

### C. Females

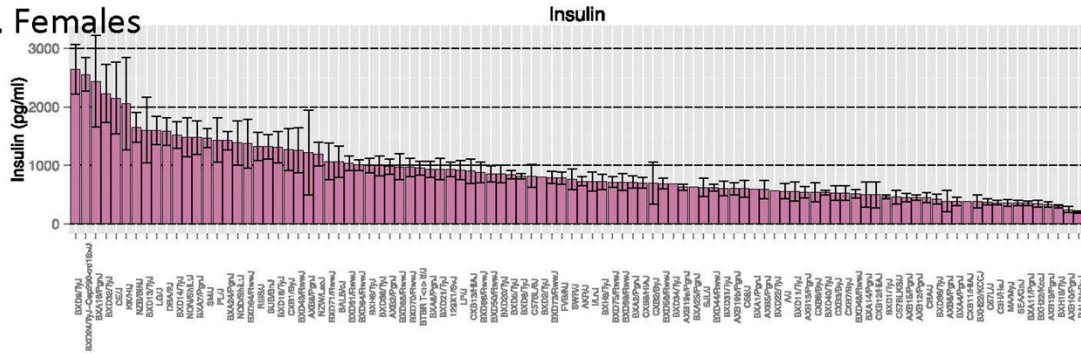

### D. Males

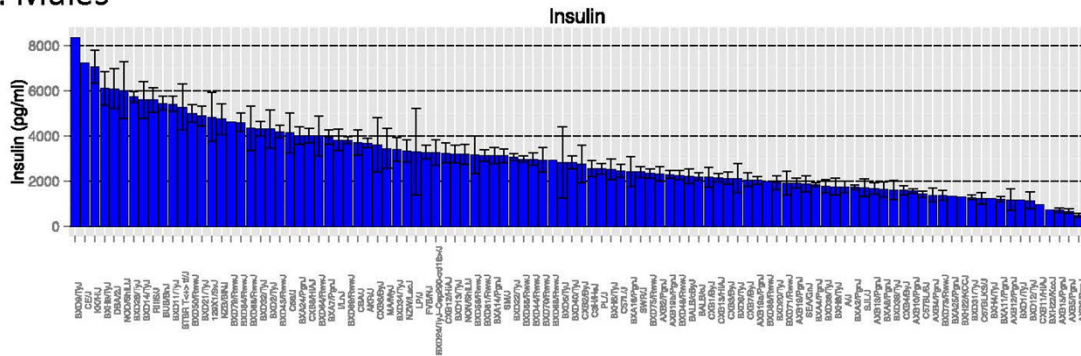

Supplement: S4 Fig — In each panel, strains are arranged in rank order by strain-average glucose or insulin levels ± SEM after 16 weeks on high-fat diet. A) Glucose (mg/dl) in females. B) Glucose (mg/dl) in males. C) Insulin (pg/ml) in females. D) Insulin (pg/ml) in males. (PDF) [file pgen.1005711.s004.pdf]

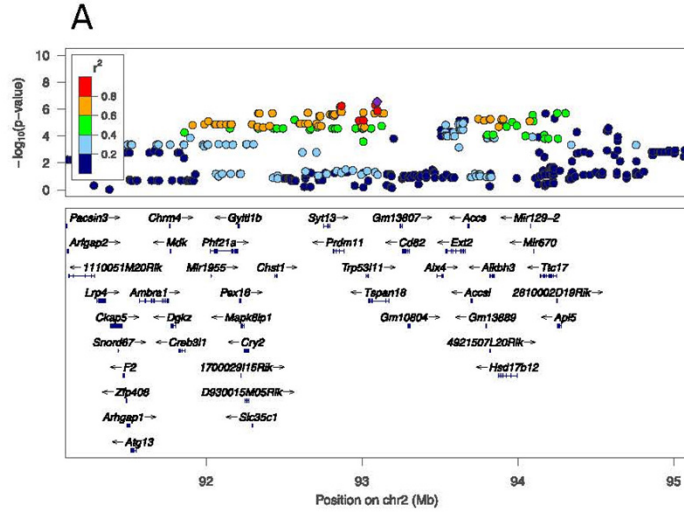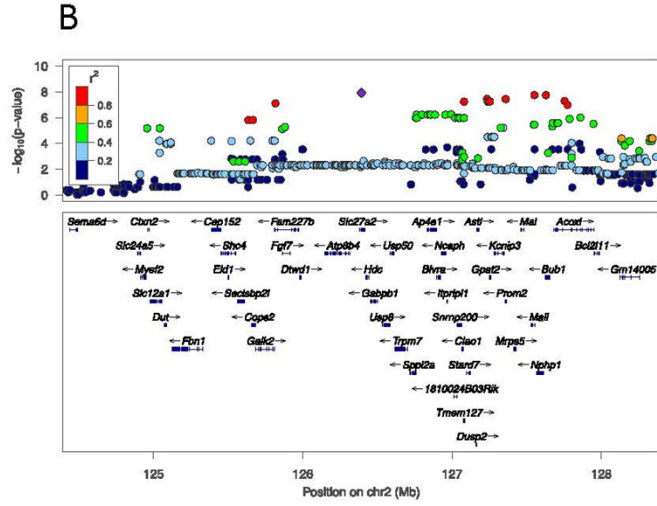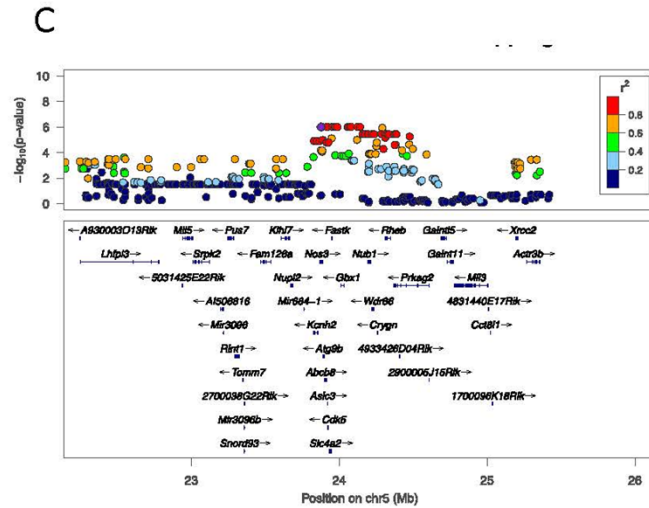

Supplement: S5 Fig — LocusZoom plots [89] of atherosclerosis QTLs on (A) Chromosome 2 at 93.3 Mb, (B) chromosome 2 at 126.6 Mb and (C) Chromosome 5 at 24.6 Mb. Significance for association of SNPs with the atherosclerosis phenotype is indicated by–log p-value. Purple diamond indicates position of peak SNP. Other SNP positions are marked as circles with color indicating R2 as shown in color scale. Positions of nearby genes are mapped below. (PDF) [file pgen.1005711.s005.pdf]

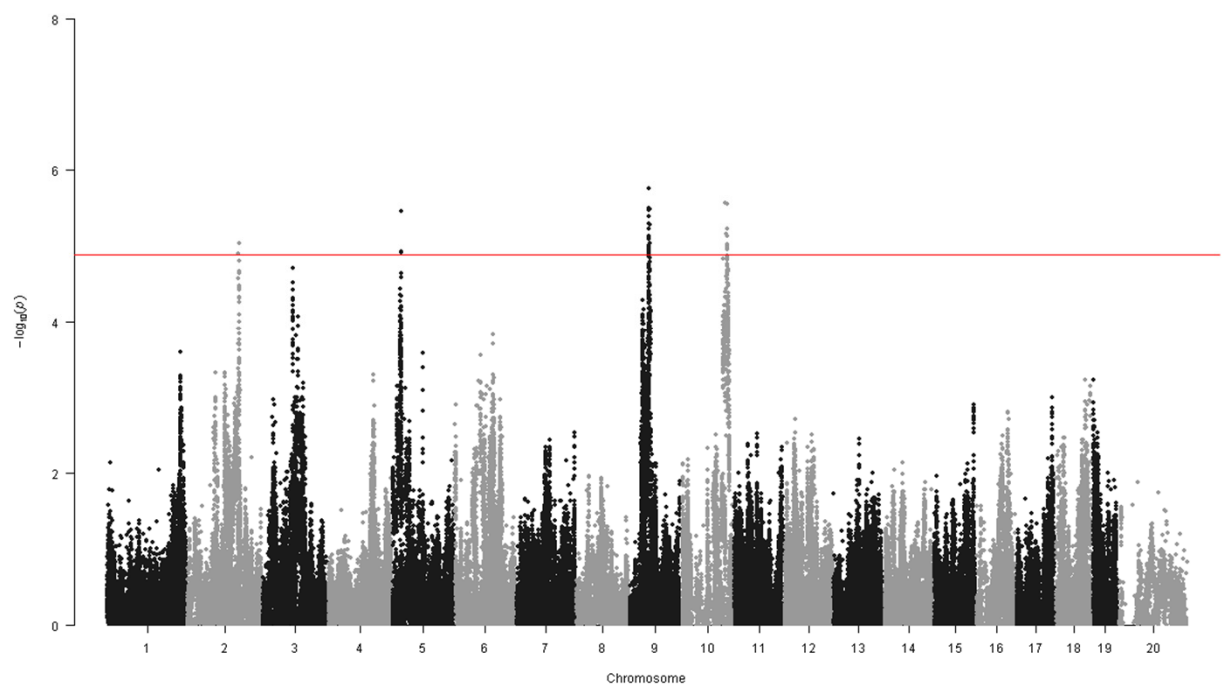

Supplement: S7 Fig — Genome wide association plot for atherosclerotic lesion-area restricted to data from strains for which 3 or more females were available. In addition to the previously observed three peaks on chromosomes 2, 5 and 9, there is an additional locus on chromosome 10 that reaches significance. (PDF) [file pgen.1005711.s007.pdf]

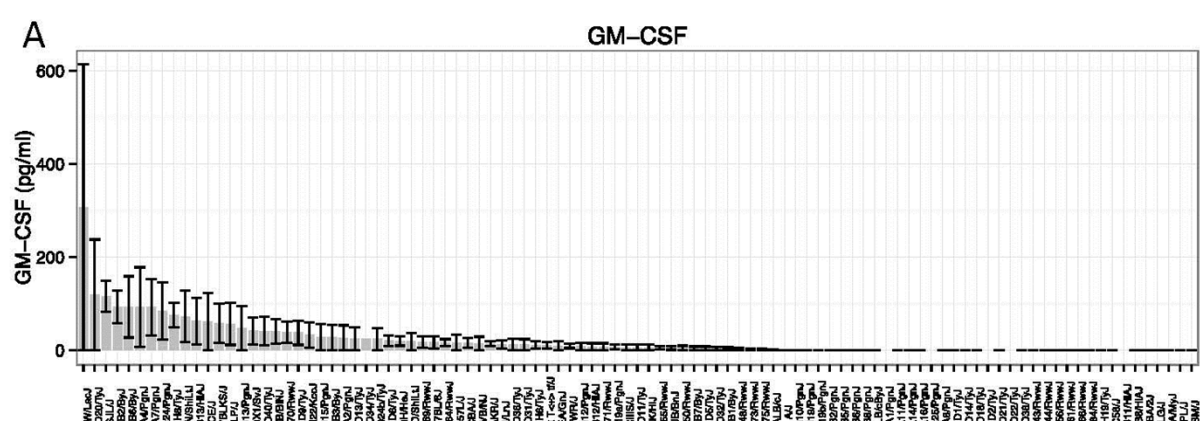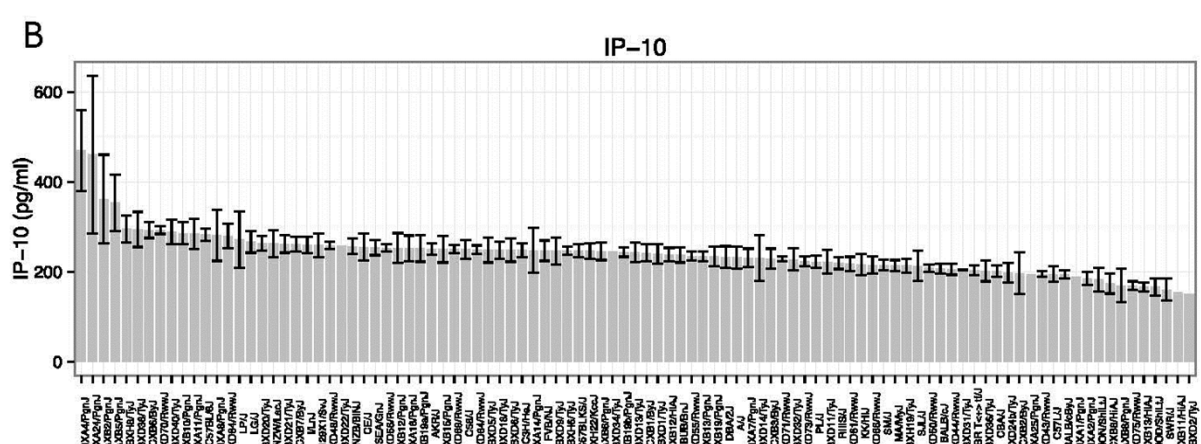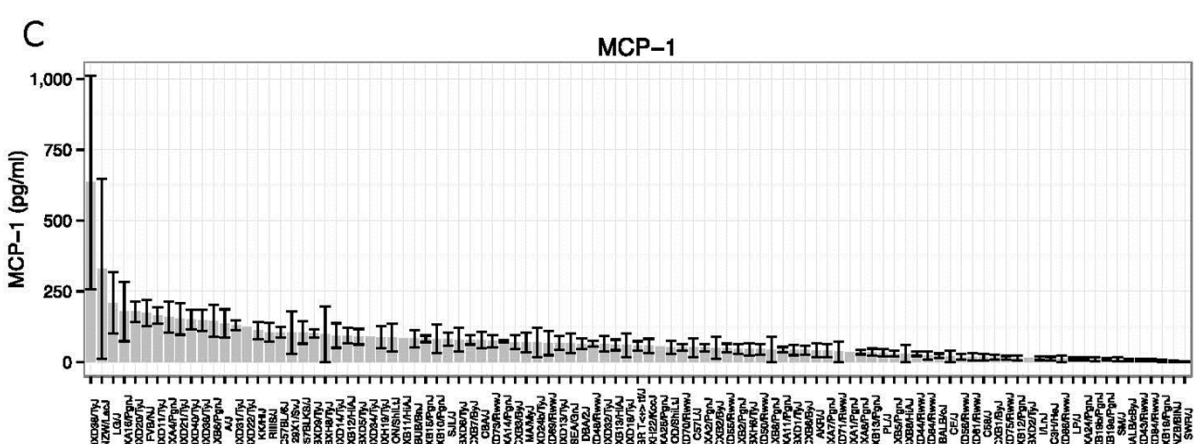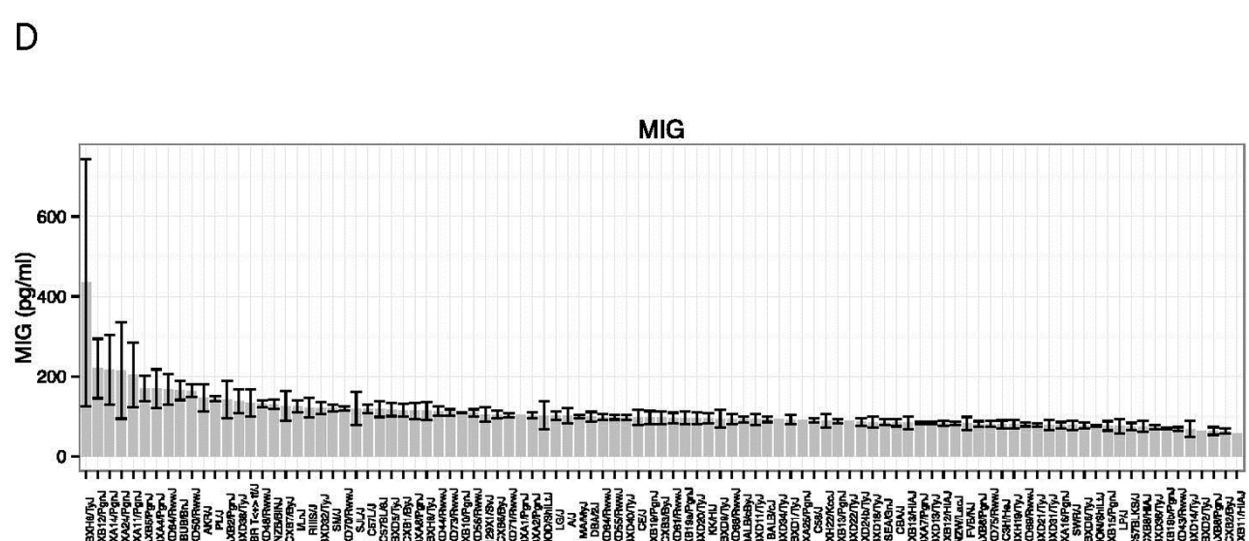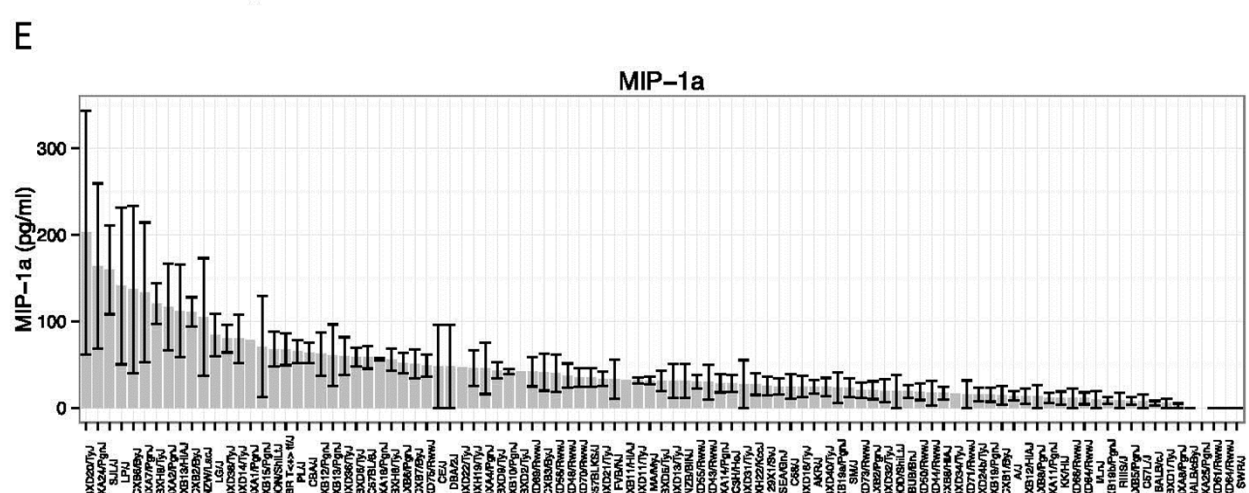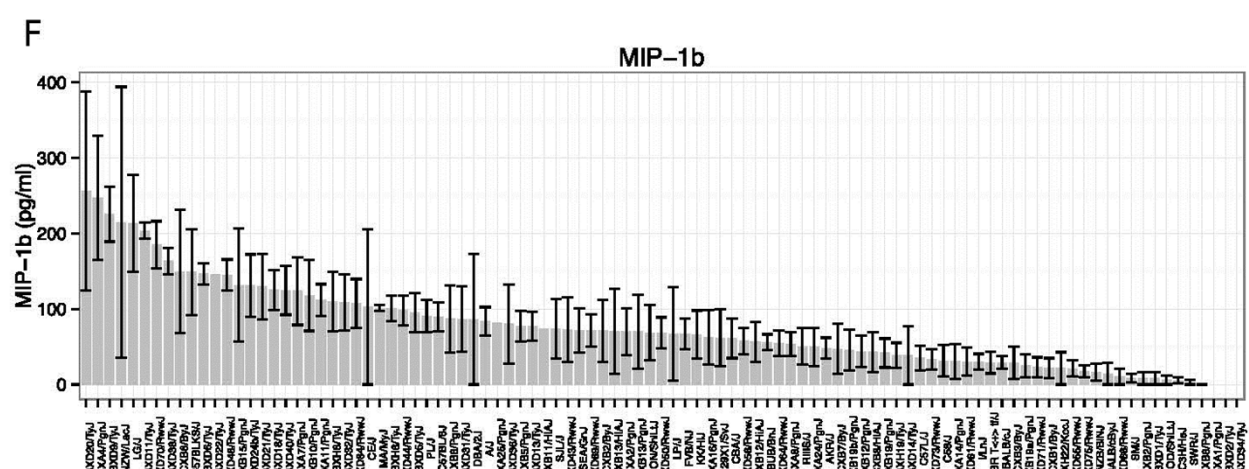

Supplement: S8 Fig — In each panel, strains are arranged in rank order by strain-average metabolite levels (pg/ml) in females +/- SEM after 16 weeks on Western Diet. (A) GM-CSF, (B) IL-10, (C) MCP-1 (D) MIG, (E) MIP-1α and (F) MIP-1β. (PDF) [file pgen.1005711.s008.pdf]

# Measurements on same or different sac dates

A

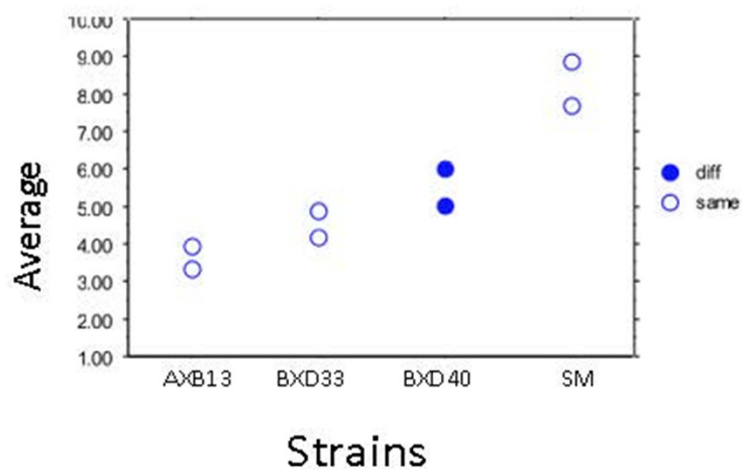

B

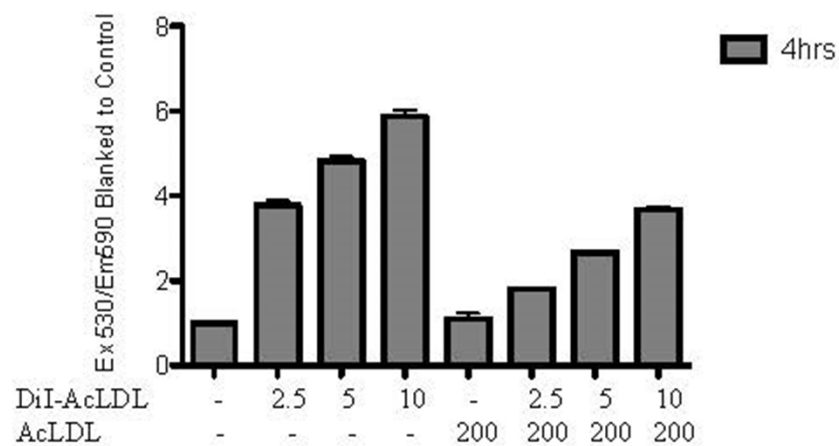

C

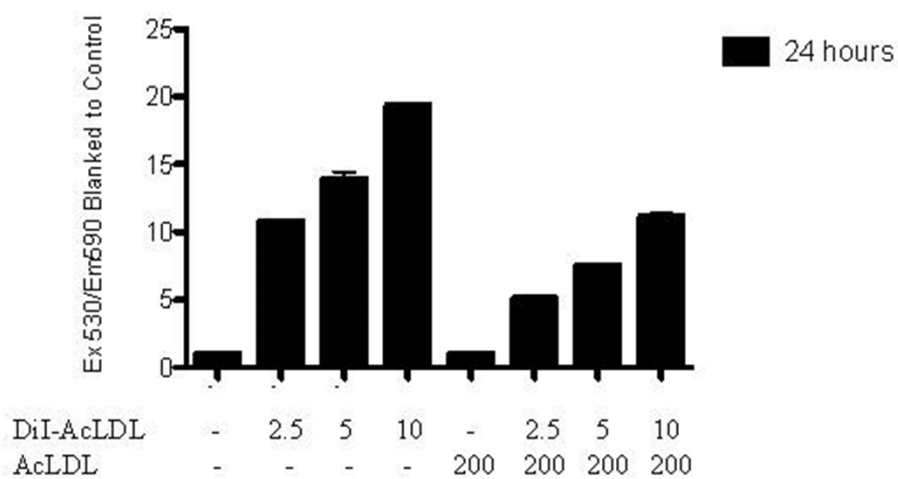

Supplement: S9 Fig — (A) Measurements on different dates for strains AXB13, BXD33, BXD40 and SM. Open circles indicate replicate mice measured on the same day. The closed circles for BXD40 indicate results for replicate mice measured on different days. (B) Relative fluorescence after varying concentrations of DiI–Ac-LDL after 4h or DiI-AcLDL plus 200 ug/ml unlabeled-AcLDL (C) Relative fluorescence after varying concentrations of DiI–Ac-LDL after 24h or DiI-AcLDL plus 200 ug/ml unlabeled-AcLDL. (PDF) [file pgen.1005711.s009.pdf]

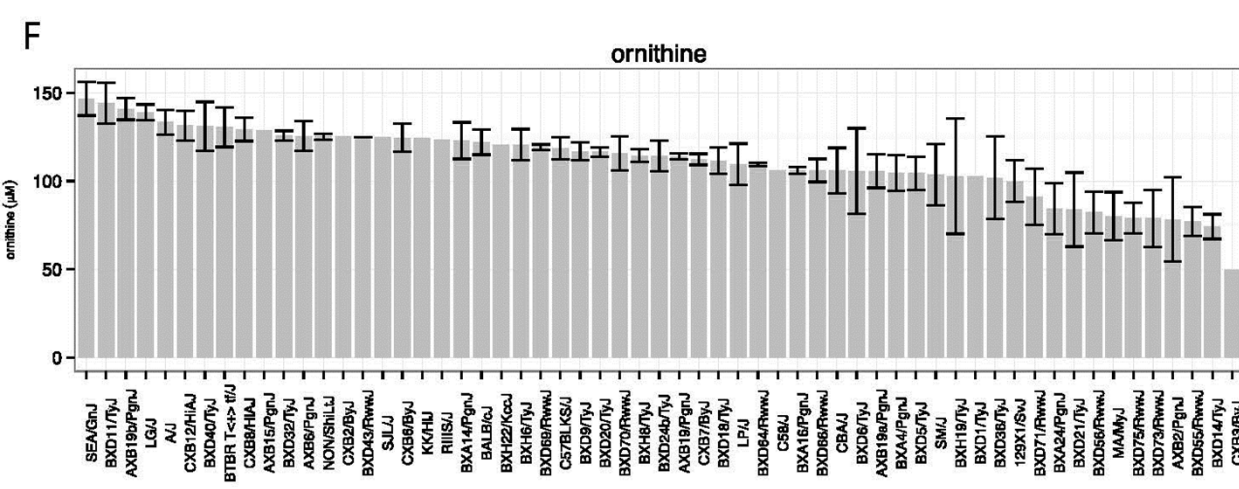

Supplement: S10 Fig — In each panel, strains are arranged in rank order by strain-average metabolite levels (uM) in females ± SEM after 16 weeks on Western Diet. (A) Butyryl-carnitine, (B) Choline, (C) Trimethylamine N-oxide (TMAO). (D) Arginine, (E) Citrulline, (F) Ornithine. (PDF) [file pgen.1005711.s010.pdf]

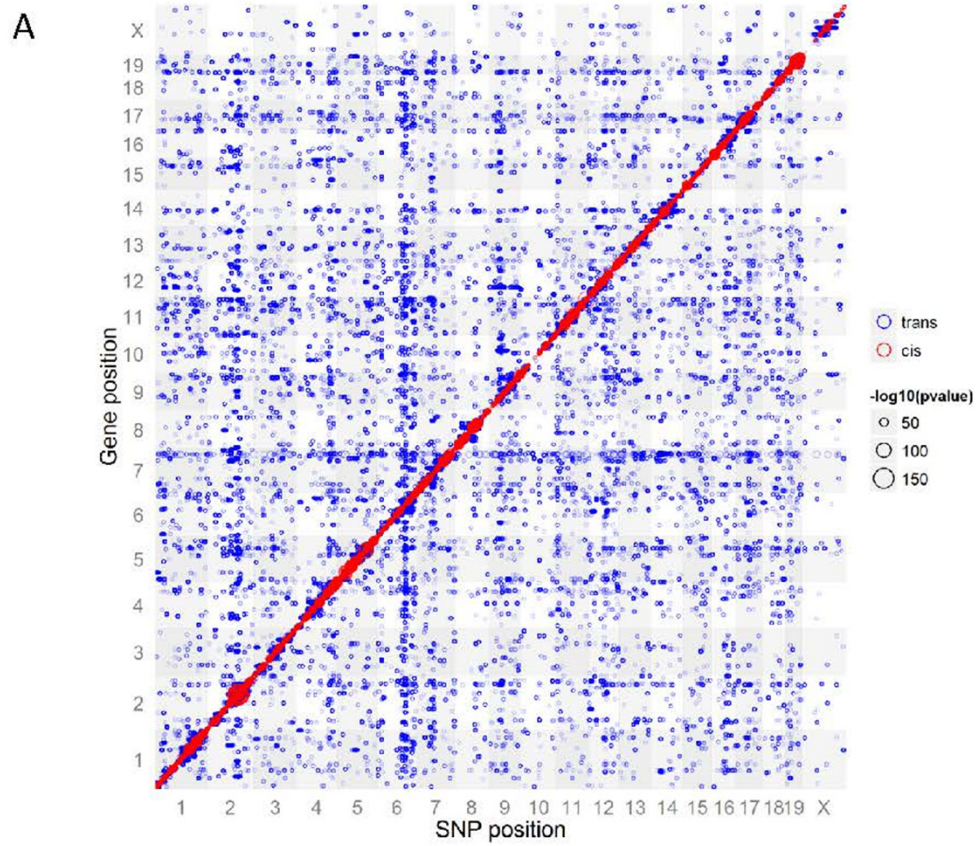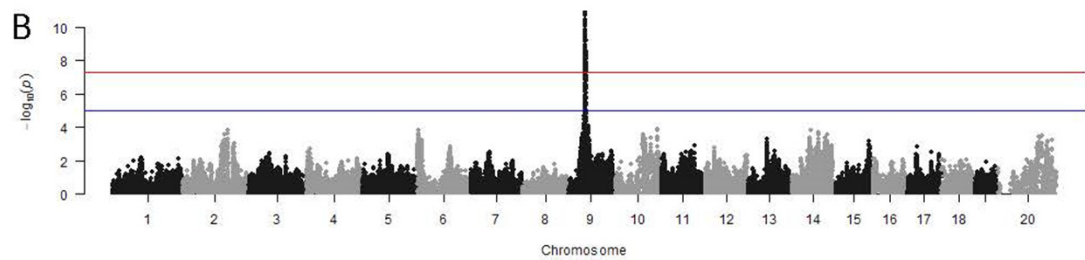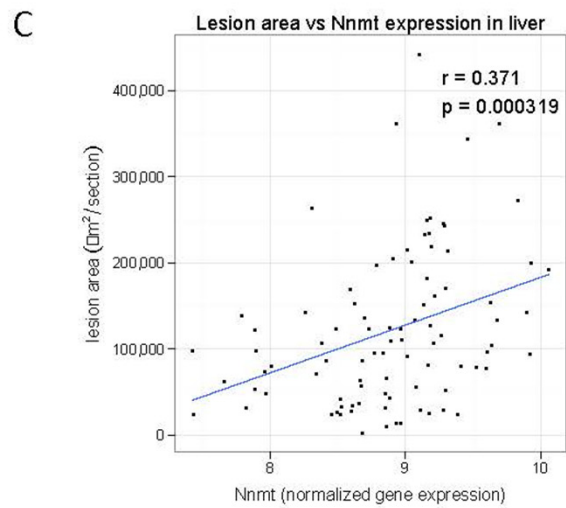

Supplement: S11 Fig — (A) Transcript levels in liver of HMDP mice were profiled and significant associations are plotted according to chromosomal position (x-axis) versus the location of the structural gene (y-axis). The strong diagonal line represents cis-eQTL, whereas the remainder are trans-eQTL signals. (B) Genome-wide association results in the HMDP demonstrating a strong association for Nnmt transcript levels in liver on chromosome 9. (C) Correlation between Nnmt mRNA levels and atherosclerosis. (PDF) [file pgen.1005711.s011.pdf]

A

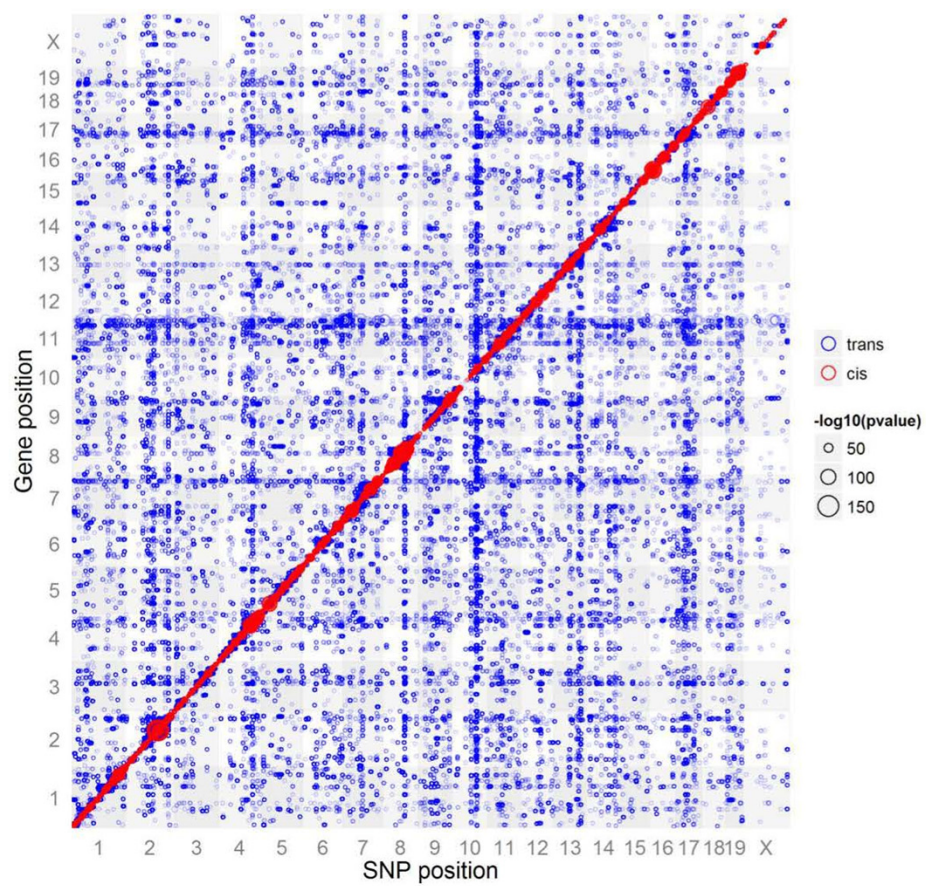

B

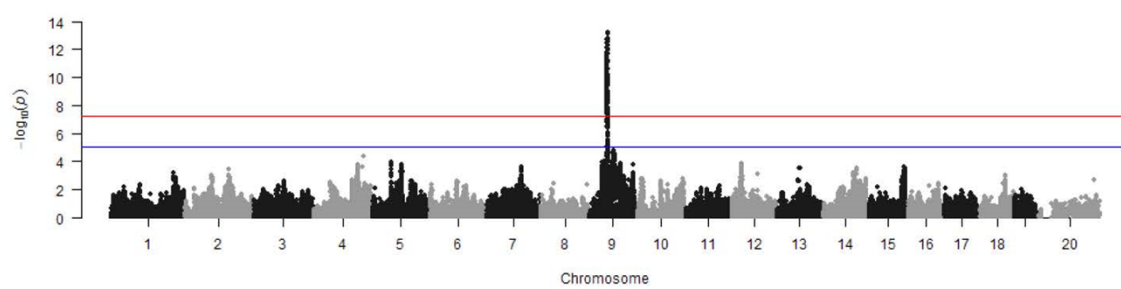

C

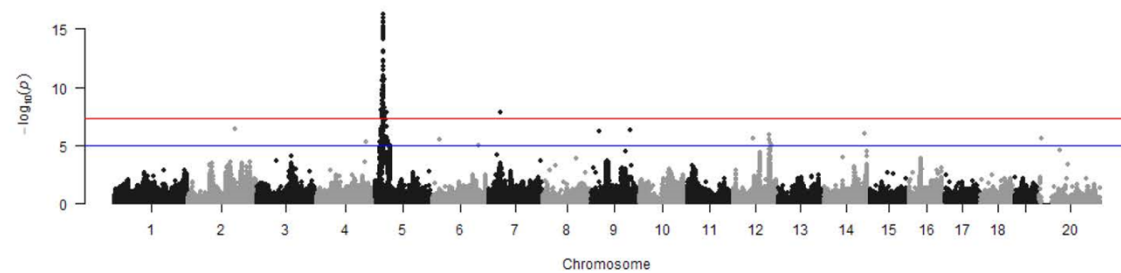

Supplement: S12 Fig — (A) Transcript levels in aorta of HMDP mice were profiled and significant associations are plotted according to chromosomal position (x-axis) versus the location of the structural gene (y-axis). The strong diagonal line represents cis-eQTL, whereas the remainder are trans-eQTL signals. (B) Genome-wide association results in the HMDP demonstrating a strong association for Nnmt transcript levels in aorta on chromosome 9. (C) Genome-wide association results in the HMDP demonstrating a strong association for Nub1 transcript levels in aorta on chromosome 5. (PDF) [file pgen.1005711.s012.pdf]
