# Supplementary material for: Genetic Architecture of Atherosclerosis in Mice: A Systems Genetics Analysis of Common Inbred Strains
Source: PLoS Genet. 2015 Dec 22;11(12):e1005711. doi: 10.1371/journal.pgen.1005711 (PMC4687930; doi:10.1371/journal.pgen.1005711)

A.

Female CHR2 Locus Conditional Analysis

Top Plot: Chr2 Mapping

Middle Plot: Conditioned on rs27381267

Bottom Plot: Conditioned on rs32754652

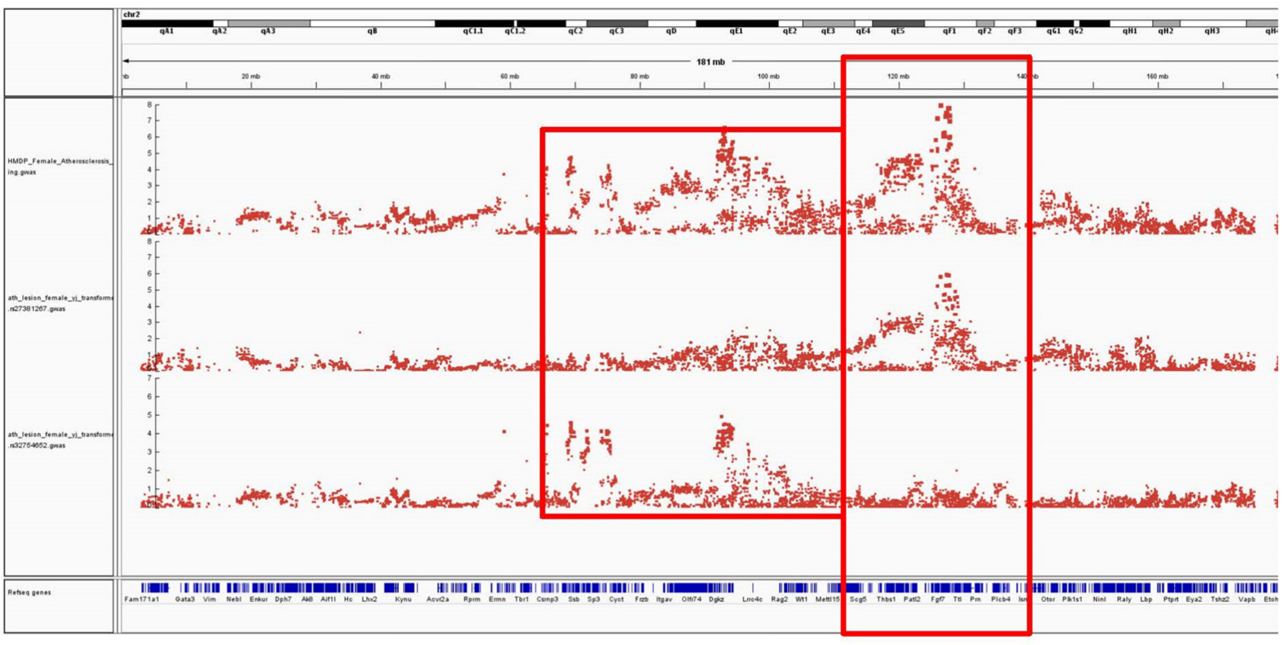

B.

Top Plot: Chr5 Mapping

Bottom Plot: Conditioned on rs32008039

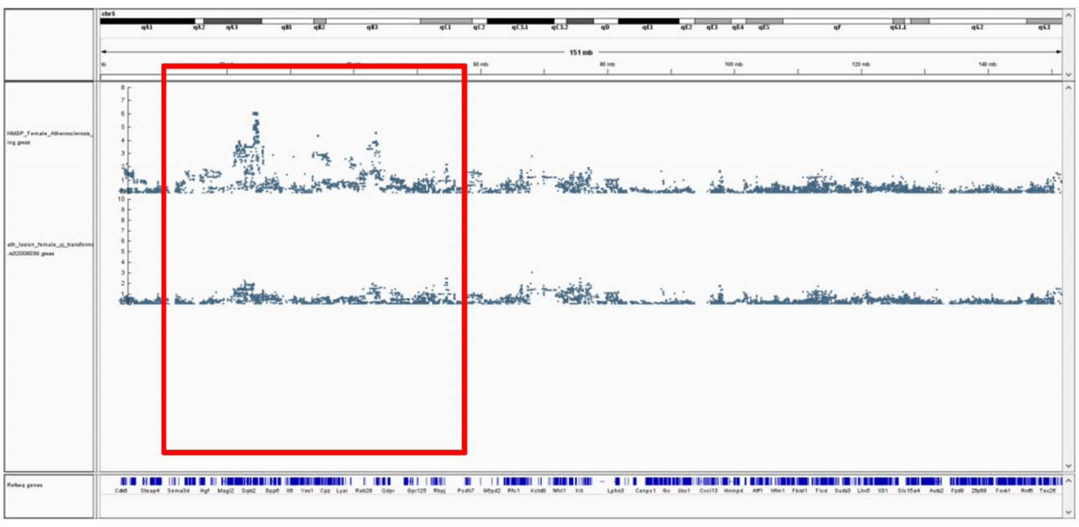

C.

Top Plot: Chr9 Mapping

Bottom Plot: Conditioned on rs33738357

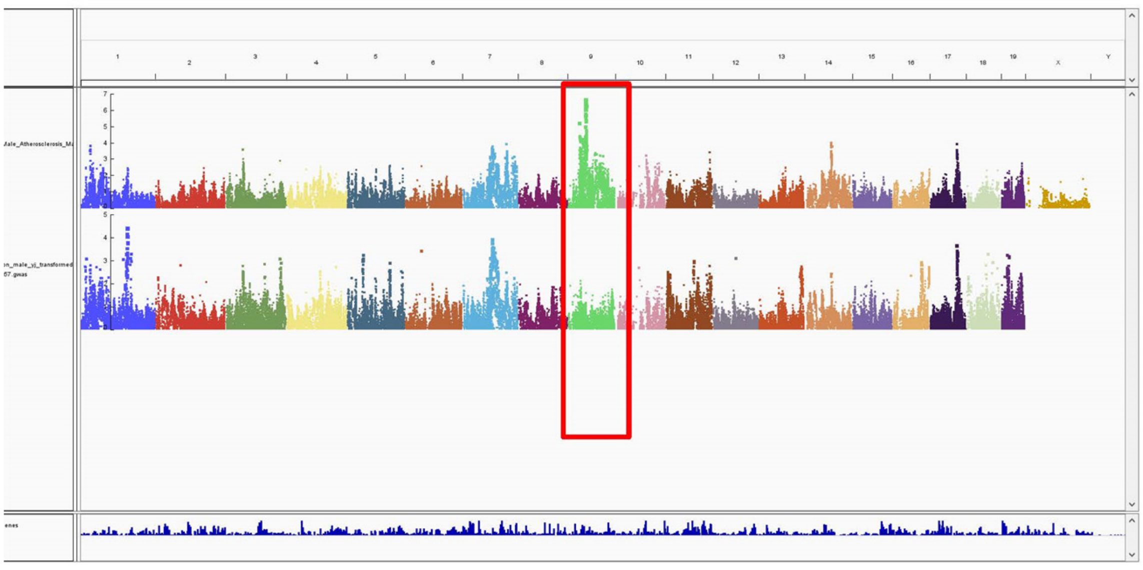

Male CHR9 Locus Conditional Analysis

D.

Female CHR9 Locus Conditional Analysis

Top Plot: Chr9 Mapping

Bottom Plot: Conditioned on rs33738357

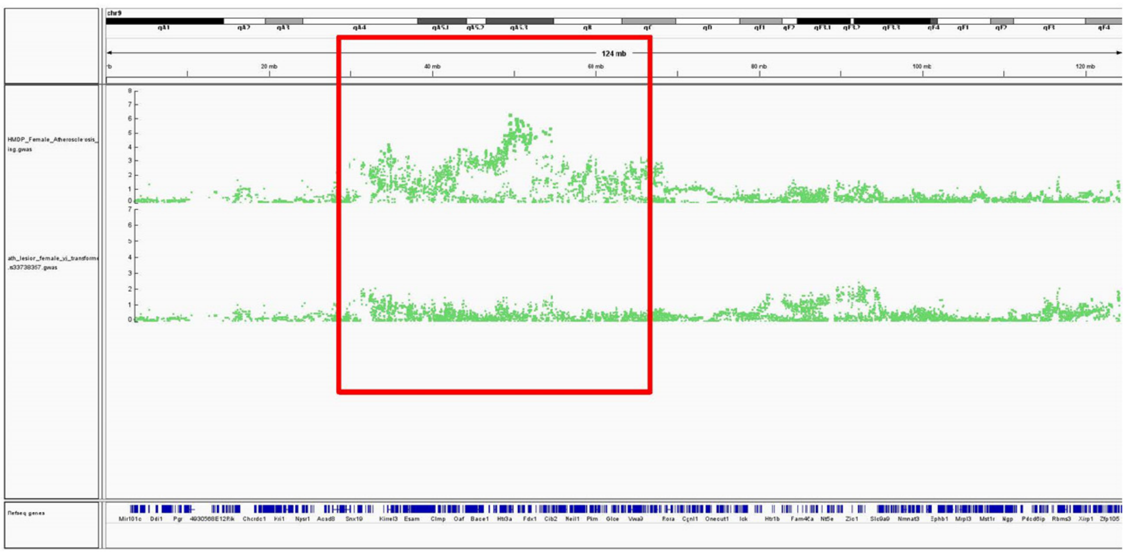

Supplement: S6 Fig — To determine if the two QTL peaks on chromosome 2 are independent, association analysis was repeated but conditioning on the peak SNP in each peak. (A) Top plot shows dual peaks in absence of conditioning. Middle plot shows association conditioned on peak SNP (rs27381267) at 93.3 Mb while bottom plot shows association conditioned on peak SNP (rs32754652) at 126.6 Mb. In each case, conditioning suppresses association for the local peak but not at the second peak, suggesting that the two association peaks are independent. (B) For QTL on chromosome 5, top plot shows broad peaks in absence of conditioning. Bottom plot shows association conditioned on peak SNP (rs32008039) at 24.6 Mb. In this case, conditioning suppresses association across the full region consistent with a single associated locus. Similarly, for QTL on chromosome 9, top plot shows broad peaks in absence of conditioning for both males (C) and females (D). Bottom plot shows association conditioned on peak SNP (rs33738357) at 49.6 Mb. Again, conditioning suppresses association across the full region consistent with a single associated locus. (PDF) [file pgen.1005711.s006.pdf]
